# MCRS1 overexpression, which is specifically inhibited by miR-129*, promotes the epithelial-mesenchymal transition and metastasis in non-small cell lung cancer

**DOI:** 10.1186/1476-4598-13-245

**Published:** 2014-11-06

**Authors:** Min-Xia Liu, Ke-Cheng Zhou, Yi Cao

**Affiliations:** Laboratory of Molecular and Experimental Pathology, Kunming Institute of Zoology, Chinese Academy of Sciences, Kunming, China; Kunming College of Life Science, University of Chinese Academy of Sciences, Kunming, China

**Keywords:** Lung cancer, MCRS1, Epithelial-mesenchymal transition, Metastasis, microRNA

## Abstract

**Background:**

Although tumor invasion and metastasis are both classical hallmarks of cancer malignancy and the major causes of poor clinical outcomes among cancer patients, the underlying master regulators of invasion and metastasis remain largely unknown. In this study, we observed that an overexpression of microspherule protein 1 (MCRS1) promotes the invasion and metastasis of non-small cell lung cancer (NSCLC) cells. Furthermore, we sought to systematically investigate the pathophysiological functions and related mechanisms of MCRS1.

**Methods:**

Retrovirus-mediated RNA interference was employed to knockdown MCRS1 expression in NSCLC cell lines. Quantitative real-time polymerase chain reaction (qRT-PCR) and western blot respectively were used to measure levels of mRNA and protein. Further cell permeability assessment, invasion and proliferation assays were conducted to evaluate MCRS1 functions *in vitro* while nude mice experiments were performed to examine metastatic capability *in vivo*. Microarray analysis and microRNA (miRNA) sequencing were respectively carried out for mRNA and miRNA expression profiling, while chromatin immunoprecipitation (ChIP), luciferase reporter assay, and miRNA transfection were used to investigate the interaction between MCRS1 and miRNAs.

**Results:**

MCRS1 knockdown induced morphological alterations, increased monolayer integrity, decreased cellular invasion and metastasis, and attenuated stemness and drug resistance among tested NSCLC cells. The levels of MCRS1 expression were likewise correlated with tumor metastasis among NSCLC patients. We identified differentially expressed genes after MCRS1 silencing, which included cell junction molecules, such as ZO-1, Occludin, E-cadherin, and DSG2. However, these differentially expressed genes were not directly recognized by a transcriptional complex containing MCRS1. Furthermore, we found that MCRS1 binds to the miR-155 promoter and regulates its expression, as well as MCRS1 promotes epithelial-mesenchymal transition (EMT), invasion, and metastasis through the up-regulation of miR-155. Systematic investigations ultimately showed that MCRS1 was directly and negatively regulated by the binding of miR-129* to its 3’-UTR, with miR-129* overexpression suppressing the growth and invasion of NSCLC cells.

**Conclusions:**

MiR-129* down-regulation induced MCRS1 overexpression, which promotes EMT and invasion/metastasis of NSCLC cells through both the up-regulation of miR-155 and down-regulation of cell junction molecules. This miR-129*/MCRS1/miR-155 axis provides a new angle in understanding the basis for the invasion and metastasis of lung cancer.

**Electronic supplementary material:**

The online version of this article (doi:10.1186/1476-4598-13-245) contains supplementary material, which is available to authorized users.

## Background

Lung cancer is one of the most common malignancies and the leading cause of cancer-related death in the world. Non-small cell lung cancer (NSCLC) comprises approximately 80% of all lung cancers. Lung cancer initiation and progression are predominately driven by genetic alterations in oncogenes and tumor suppressor genes [1]. Although significant progress had been made in the discovery of lung cancer-related genes, the pathophysiological functions and mechanisms underlying these aberrant genes remain unclear. In previous studies, we observed a chromosome 12q13 abnormality [2] and further investigated the expression of 20 genes within the 12q13 region, observing the overexpression of microspherule protein 1 (MCRS1) in NSCLC cells [3]. MCRS1, also known as p78 and MSP58, was initially identified as an interacting partner of nucleolar protein p120 [4]. MCRS1 participates in the regulation of gene transcription by interacting with various proteins, including nucleolar proteins, transcription factors, and RNA-binding proteins [4–7]. TOJ3 (a quail homolog of MCRS1) behaves as an oncogene during fibroblast transformation, and this transformation activity can be suppressed by the tumor suppressor PTEN [8, 9]. MCRS1 overexpression has been documented in a variety of human cancers, and this overexpression was found to induce the proliferation of cancer cells [3, 10–12]. In the present study, we observed that MCRS1 overexpression promoted the epithelial-mesenchymal transition (EMT) and metastasis in NSCLC cells.

EMT and its reverse process, the mesenchymal-epithelial transition (MET), are key programs in embryogenesis. During the EMT process, epithelial cells lose their characteristics, such as cell-cell contacts and polarity, and adopt a mesenchymal phenotype of increased migratory behavior. Over the past decades, growing evidence has demonstrated that EMT contributes to cancer development and metastasis [13]. Moreover, EMT can result in drug resistance in cancer cells and the acquisition of stem-like cells [14]. The molecular mechanisms of EMT involve many aspects, including transcription factors, cellular junctions, cytoskeleton, and microRNAs (miRNAs). Here, we explore the mechanisms by which MCRS1 contributes to the EMT program.

MCRS1 overexpression has been documented in a variety of human tumors [3, 10–12]; however, the molecular mechanisms underlying MCRS1 alteration remain elusive. In this study, we investigated the mechanisms of MCRS1 regulation in NSCLC cells.

## Results

### MCRS1 depletion reverses EMT in lung cancer cells

The levels of MCRS1 expression were first determined in 7 lung cancer cell lines and 16HBE. There is no significant difference in the expression of MCRS1 among the 7 NSCLC cell lines (Additional file 1a). EPLC-32 M1 and NCI-H292 that displayed very high levels of MCRS1 protein, were used for MCRS1 knockdown. We stably reduced the MCRS1 expression in the EPLC-32 M1 and NCI-H292 using RNA interference mediated by a retroviral system (pSIREN-RetroQ), and the decreased expression of MCRS1 was examined using quantitative Real-Time Polymerase Chain Reaction (qRT-PCR) and western blot (Additional file 1b, c).

**Figure 1:**
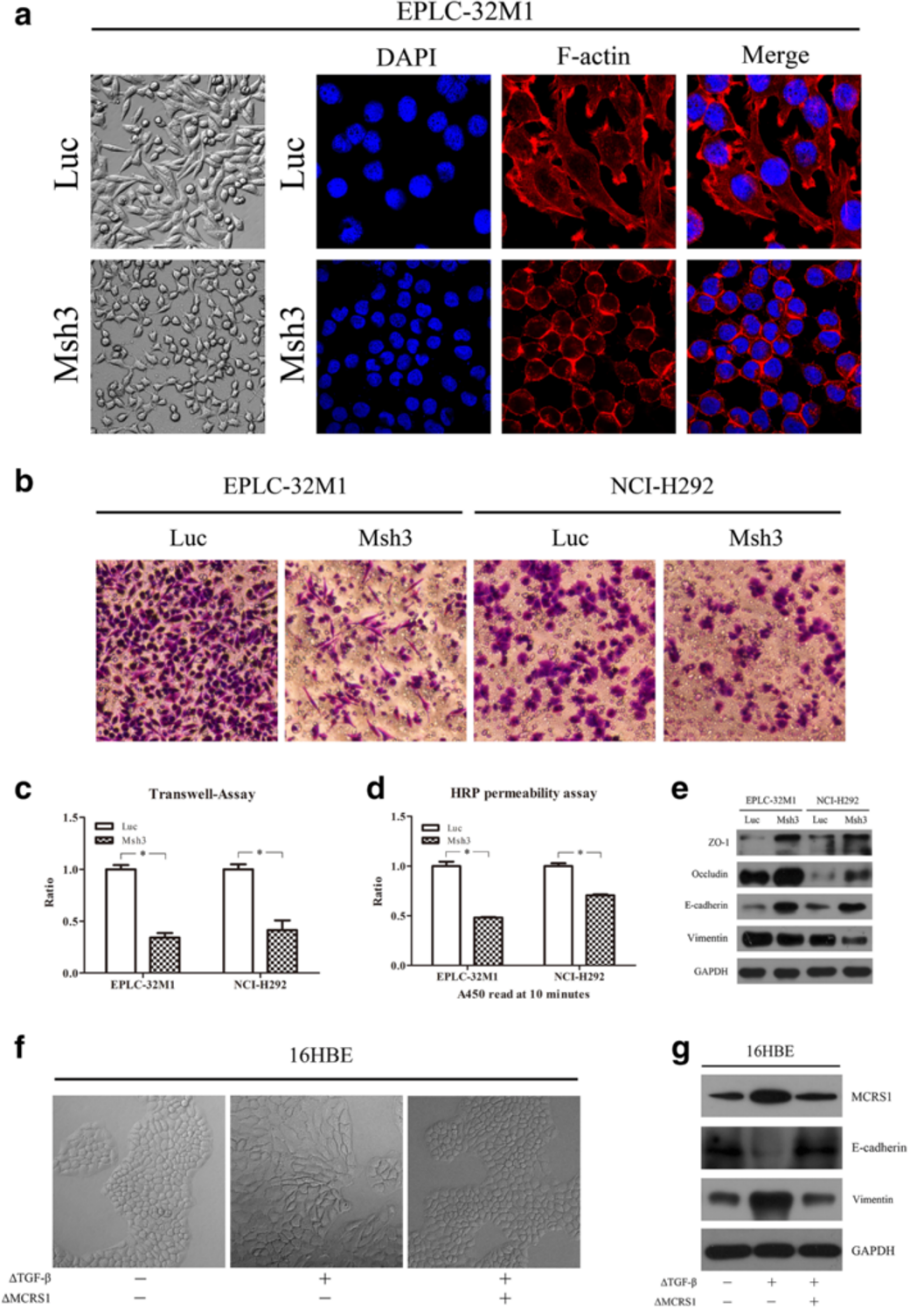
**Results of the morphological and functional experiments in NSCLC cells after MCRS1 silencing. (a)** Left: Morphological observation of EPLC-32 M1 cells using phase-contrast microscopy. Right: Images of F-actin stained EPLC-32 M1 cells. **(b and c)** The results of the matrigel invasion assays in EPLC-32 M1 and NCI-H292 cells with or without MCRS1 knockdown. The invaded cells are stained with crystal violet **(b)**, and the fold change in the number of invading cells is given as the mean ± SD from three independent experiments **(c)**. **(d)** Cell permeability assay of the EPLC-32 M1 and NCI-H292 cells with or without MCRS1 silencing. The values of HRP permeability are given as the mean ± SD. **(e)** Western blot analysis of ZO-1, Occludin, E-cadherin and Vimentin in EPLC-32 M1 and NCI-H292 cells with or without MCRS1 depletion. **(f)** Morphological changes in 16HBE cells treated by TGF-β (ΔTGF-β) after transient MCRS1 knockdown (ΔMCRS1). **(g)** Western blot analysis of MCRS1, E-cadherin,and Vimentin in 16HBE cells treated by TGF-β (ΔTGF-β) after transient MCRS1 knockdown (ΔMCRS1). Luc: cells without MCRS1 silencing; Msh3: cells with stable MCRS1 silencing; *P <0.05 (Student’s t-test).

Characteristic MET morphological changes were observed in the EPLC-32 M1 after MCRS1 depletion, whereby the typical spindle-like appearance of fibroblast cells was replaced with the round shape, epithelial morphology under phase-contrast microscopy (Figure 1a Left). Following TRITC-phalloidin staining for F-actin, the MCRS1-silenced cells exhibited cortical bundles compared to the control cells, which displayed contractile bundles, indicating that MCRS1 overexpression could affect cytoskeletal rearrangement (Figure 1a Right). In the meantime, reduced cell size was observed in MCRS1-depleted cells.

As dramatic changes in cell shape were observed after MCRS1 depletion, we assessed the influence of MCRS1 on cellular invasion and junctions. MCRS1 suppression significantly reduced cell invasion, as assessed using a Transwell invasion assay (Figure 1b, 1c). Additionally, to measure the junction integrity, we performed cell permeability assays based on the Boyden chamber using horseradish peroxidase (HRP) as a tracer [15]. Silencing MCRS1 in the EPLC-32 M1 and NCI-H292 significantly reduced the permeability of the cells to HRP, indicating that MCRS1 overexpression could disrupt the functions of tight junctions (TJs) (Figure 1d). Furthermore, we examined key molecules related to EMT characteristics at the protein level. As shown in Figure 1e, the cells depleted of MCRS1 had higher levels of epithelial markers such as ZO-1, Occludin, and E-cadherin, as well as a lower level of Vimentin (mesenchymal maker).

To confirm the role of MCRS1 in the EMT program, we chose the 16HBE cells with the lowest level of MCRS1 expression as an *in vitro* model, and then treated 16HBE with TGF-β1, the main inducer of EMT [13]. As anticipated, the induced cells acquired the appearance of mesenchymal-like cells, exhibited the increased expression of MCRS1 and Vimentin as well as the reduced expression of E-cadherin (Figure 1f, 1g). Additionally, we performed MCRS1 knockdown in TGF-β1 treated cells, and found that MCRS1-shRNA depletion could reverse functions of TGF-β1 treatment and lead to an increased expression of E-cadherin and a decreased expression of Vimentin (Figure 1g). These results indicated that MCRS1 deregulation may be involved in the EMT program.

Taken together, the changes in cellular morphology, permeability, and invasion and alterations in the expression of EMT-related molecules after MCRS1 silencing demonstrated that MCRS1 could contribute to the EMT program in NSCLC cells.

### The down-regulation of MCRS1 attenuates drug resistance and the generation of CSC-like cells from NSCLC cells

**Figure 2:**
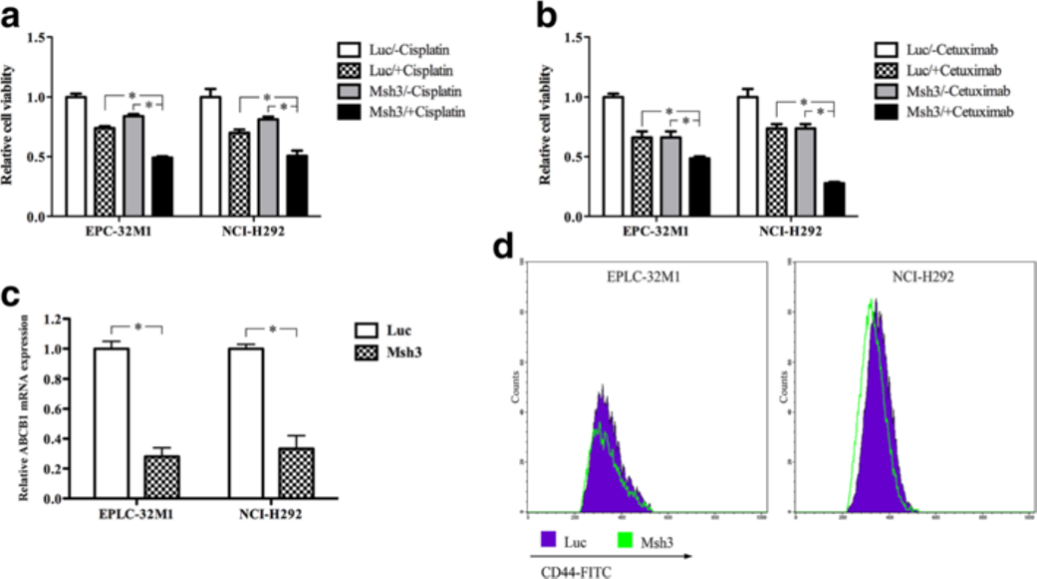
**The drug resistance and generation of CD44** ^**+**^ **CSC-like cells in cultured NSCLC cells after MCRS1 silencing. (a)** Assessment of the viability of EPLC-32 M1 and NCI-H292 cells after cisplatin treatment for 72 h. **(b)** Assessment of the viability of EPLC-32 M1 and NCI-H292 after cetuximab treatment for 72 h. **(c)** The expression of ABCB1 mRNA after MCRS1 depletion. **(d)** Flow cytometric analysis of CD44 expression in EPLC-32 M1 and NCI-H292 cells with or without MCRS1 knockdown. Luc: cells without MCRS1 silencing; Msh3: cells with stable MCRS1 silencing. *P <0.05 (one-way ANOVA and Student’s t-test).

As shown in Figure 2a and 2b, compared with MCRS1 depletion alone (no drug treatment) and the drug treatments alone (no MCRS1 depletion), MCRS1 silencing significantly inhibited the growth of EPLC-32 M1 and NCI-H292 after treatments with cisplatin (a common chemotherapy drug for NSCLC treatment) and cetuximab (a humanized anti-EGFR antibody used to treat advanced lung cancer). Furthermore, MCRS1 suppression significantly decreased mRNA expression of ABCB1 (multidrug resistance gene, Figure 2c) [16]. Collectively, these observations indicated that MCRS1 overexpression could trigger drug resistance.

We also analyzed the expression of the putative cancer stem cell (CSC) marker CD44 [17]. As determined by flow cytometric analysis, both EPLC-32 M1 and NCI-H292 exhibited lower levels of CD44 expression after MCRS1 silencing (Figure 2d).

### MCRS1 silencing inhibits tumor metastasis in an experimental animal model

**Figure 3:**
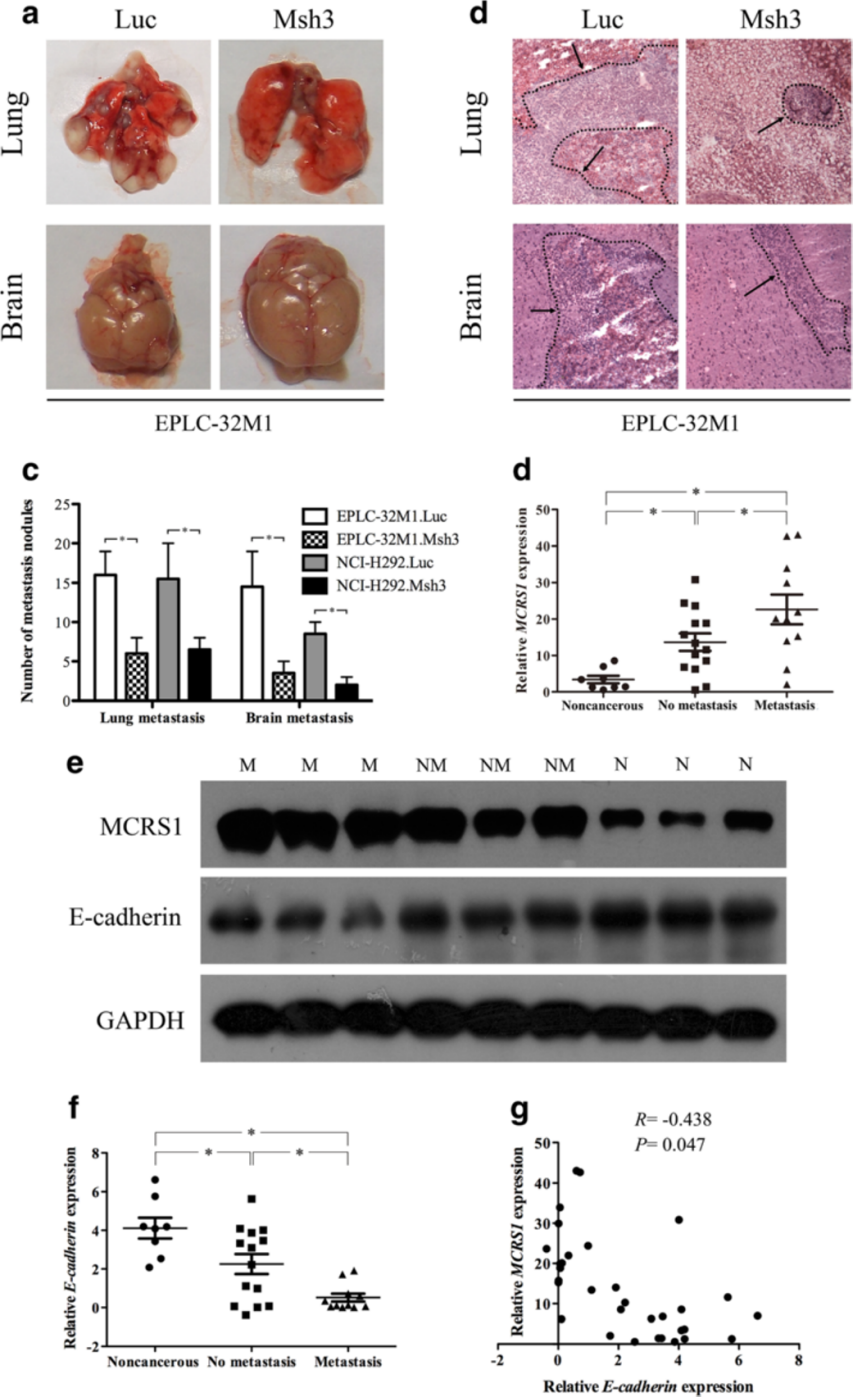
**MCRS1 expression promoted tumor metastasis in NSCLC cells.** a-c: Assessment of tumor metastasis of EPLC-32 M1 cells with and without MCRS1 silencing in nude mice. **(a)** Morphological observation of lung and brain metastases. **(b)** Histopathological observation of representative lung and brain sections, arrow: metastatic cancer cells. **(c)** The number of metastatic nodules in the lung and brain were counted and analyzed. **(d)** The relative expressions of MCRS1 mRNA in three groups of 33 tissue sample, including 8 noncancerous tissues, 14 NSCLCs without metastasis, and 11 NSCLCs with metastasis. **(e)** The expression of MCRS1 and E-cadherin proteins in clinical samples. N: Noncancerous; NM: No metastasis; M: Metastasis. **(f)** The relationship between the E-cadherin mRNA expression and the status of tumor metastasis. **(g)** The inverse correlation relationship between the MCRS1 and E-cadherin expression (Pearson’s method, R = -0.438). Luc: cells without MCRS1 silencing; Msh3: cells with stable MCRS1 silencing. Noncancerous: patient with no cancer. No metastasis: patients without any metastases. Metastasis: patients with lymph node metastases and/or blood spread. *P <0.05 (one-way ANOVA and Student’s t-test).

To determine whether the abnormal expression of MCRS1 could initiate tumor metastasis *in vivo*, MCRS1-depleted cells and control cells were injected into nude mice through the lateral tail vein. As shown in Figure 3a, the mice injected with the MCRS1-depleted EPLC-32 M1 displayed a small number of visible metastatic nodules in the lung and brain compared to the corresponding control group. The metastases in the lung and brain were confirmed by histological examination (Figure 3b), and a statistical analysis revealed that MCRS1 silencing dramatically attenuated the ability of the cancer cells to form metastases in the lung and brain (Figure 3c). Similar results were also found in the NCI-H292 after MCRS1 knockdown. Taken together, these findings demonstrated that MCRS1 overexpression strongly promoted the metastasis of NSCLC cells *in vivo*.

### The mRNA levels of MCRS1 are associated with tumor metastasis in NSCLC patients

To investigate whether MCRS1 overexpression clinically correlated with the metastatic capacity of NSCLC cells, we conducted quantitative experiments (qRT-PCR) to examine the MCRS1 expression level in 33 human tissue samples, including 25 NSCLC samples (14 tumors without metastasis and 11 tumors with lymph node and/or blood metastasis) and 8 noncancerous lung samples. The statistical analysis revealed that the MCRS1 mRNA levels in the tumors with metastases were higher than in the metastasis-free tumors or the noncancerous lung samples (Figure 3d). Similar as the MCRS1 mRNA levels, the levels of MCRS1 protein were also significantly higher in NSCLCs with metastasis compared with NSCLCs without metastasis by Western blot analysis (Figure 3e). Furthermore, we examined levels of E-cadherin mRNA and protein in human specimens, and found that values of E-cadherin in NSCLCs with metastases were markedly lower than in NSCLCs without metastases (Figure 3e, 3f). The correlation analysis revealed a negative relationship between MCRS1 and E-cadherin expressions in NSCLC tissues (Figure 3g). Collectively, MCRS1 expressions were associated with the status of tumor metastasis in clinical samples.

### The mRNA expression scan identified differentially expressed genes after MCRS1 knockdown

**Figure 4:**
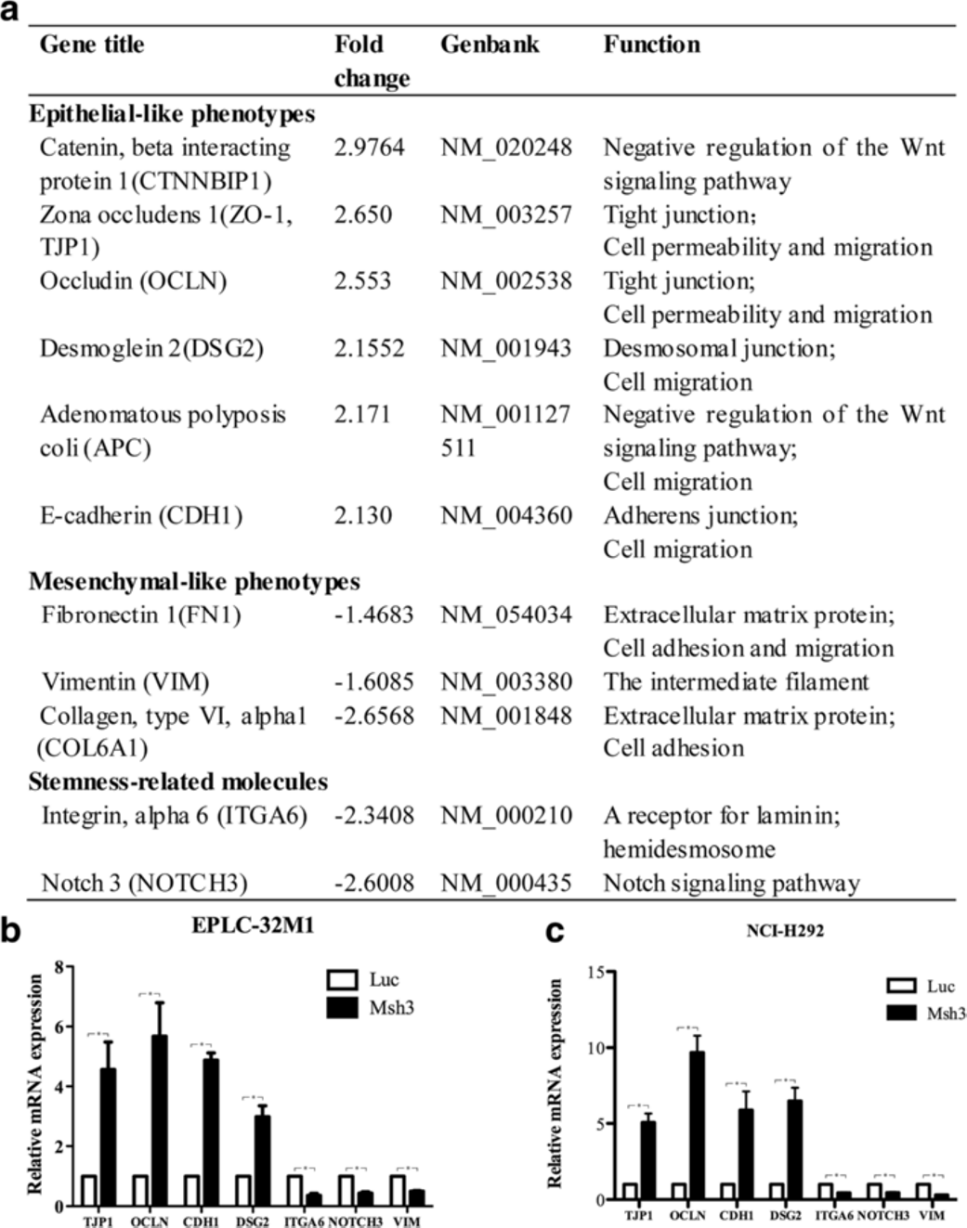
**Differentially expressed genes related to EMT process after MCRS1 silencing. (a)** A mRNA microarray analysis of the expression changes of genes related to EMT in EPLC-32 M1 cells following stable MCRS1 knockdown. +, up-regulation; -, down-regulation. **(b, c)** qRT-PCR validation of differentially expressed genes with and without MCRS1 knockdown in EPLC-32 M1 and NCI-H292 cells. Luc: cells without MCRS1 silencing; Msh3: cells with stable MCRS1 silencing. The results were analyzed by student’s t-test (P <0.05).

To explore the molecular effectors associated with MCRS1 activity, we performed a microarray analysis to determine the mRNA profiles of EPLC-32 M1 following MCRS1 knockdown. The results revealed that 237 and 132 genes were up-regulated and down-regulated, respectively, after MCRS1 silencing. Through a bioinformatics analysis using Gene Ontology (GO) terms, the genes regulated by MCRS1 were grouped into several categories (Additional file 2). Additionally, using a Kyoto Encyclopedia of Genes and Genomes (KEGG) analysis we were particularly interested in how cell junctions and Notch signaling were affected by MCRS1 due to their potential roles in EMT and tumor metastasis (Figure 4a, Additional file 3). The expression levels of EMT-related molecules, such as ZO-1 (TJP1), Occludin (OCLN), desmoglein 2 (DSG2), E-cadherin (CDH1), ITGA6, Notch 3 (NOTCH3) and Vimentin (VIM), were verified by qRT-PCR (Figure 4b, 4c). Subsequently, we investigated the relationship between MCRS1 and its downstream molecules (ZO-1, Occludin, E-cadherin, DSG-2). Through bioinformatics analysis and prediction, MCRS1 could not directly bind the promoters of these downstream molecules. Using chromatin immunoprecipitation (ChIP) assay, we also did not find that MCRS1 bound directly the promoters of these genes (Additional file 4). Based on these data, MCRS1 may regulate the expressions of the genes related to cell junctions and the Notch pathway, however, those genes were not directly recognized by a transcriptional complex containing MCRS1.

### MCRS1 significantly increase the expression of miR-155 in NSCLC cell lines

**Figure 5:**
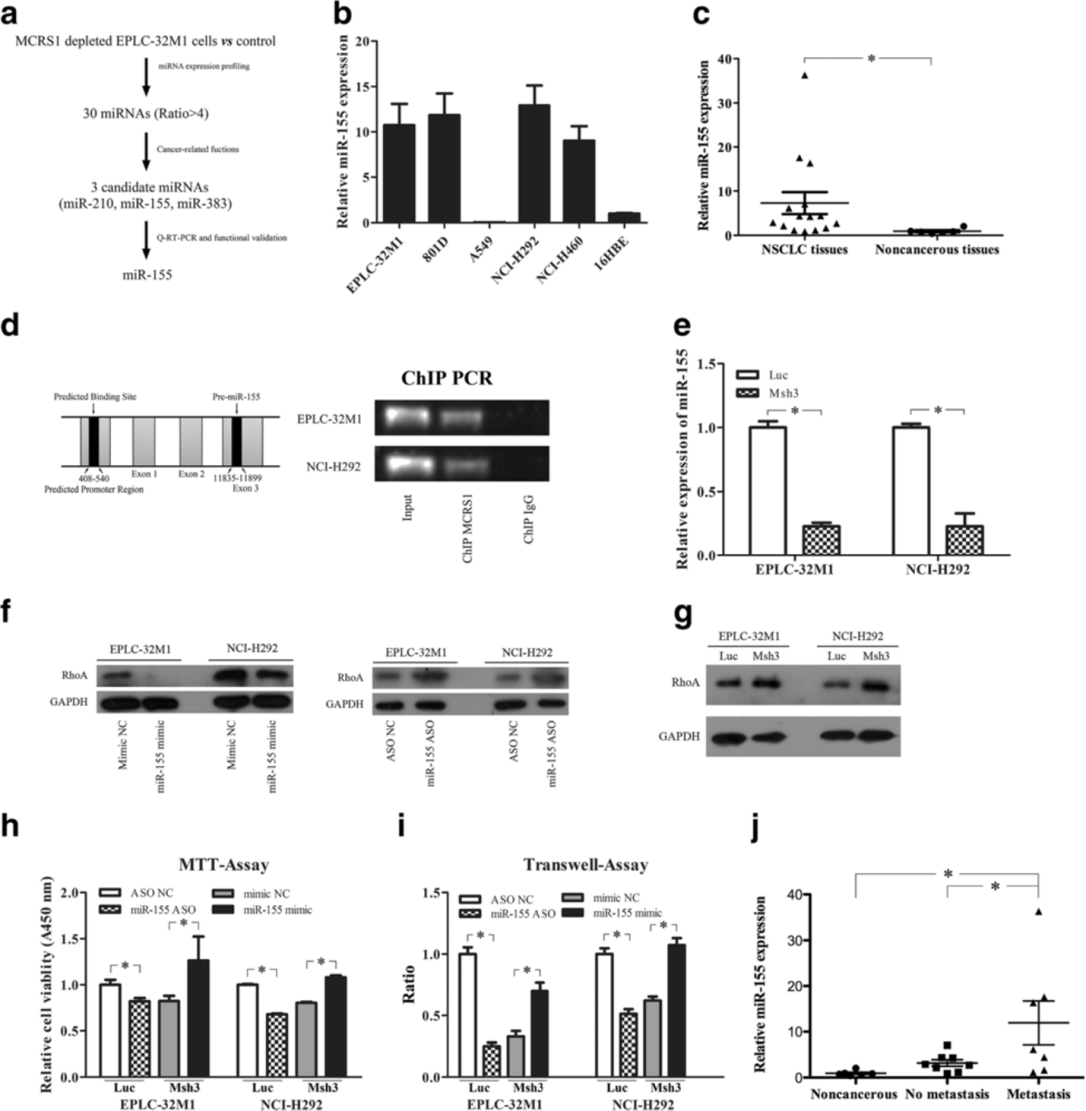
**MCRS1 promoted EMT through the up-regulation of miR-155. (a)** Screening strategy of miR-155. **(b)** The relative expression of miR-155 in NSCLC cell lines. **(c)** The level of miR-155 in clinical samples including 6 noncancerous lung tissue and 15 NSCLC tissues (Student’s t-test, *P <0.05). **(d)** Left: The map of miR-155 host gene (MIR155HG). Right: miR-155 was identified as a downstream target of MCRS1 using ChIP analysis; normal mouse IgG was used as the immunoprecipitation negative control. Input and immunoprecipitated DNAs were analyzed by PCR using primers specific for the promoter of the miR-155 host gene (MIR155HG). **(e)** miR-155 expression in EPLC-32 M1 and NCI-H292 cells with and without MCRS1 silencing. **(f)** Protein expression of RhoA (a target of miR-155) in EPLC-32 M1 and NCI-H292 cells after miR-155 treatment. **(g)** Protein expression of RhoA in EPLC-32 M1 and NCI-H292 cells with and without MCRS1 silencing. **(h, i)** The miR-155 inhibitor (miR-155 ASO) treatment decreased cell growth and invasion in cancer cells without MCRS1 silencing, and the miR-155 mimic treatment rescued the effects of MCRS1 with regard to cell growth and invasion in MCRS1–depleted cells. **(j)** The relationship between the level of miR-155 expression and the status of tumor metastasis (*P <0.05; one-way ANOVA and Student’s t-test). Luc: cells without MCRS1 silencing; Msh3: cells with stable MCRS1 silencing; NC: negative control. ASO: antisense oligonucleotide. Noncancerous: noncancerous lung tissues; No metastasis: NSCLCs without metastasis; Metastasis: NSCLCs with metastasis.

MiRNAs are small non-coding RNAs that regulate gene expression via either translation inhibition or mRNA degradation [18], and have been shown to have both pro- and anti-metastatic effects [18–20]. To gain further insight into whether MCRS1 regulates EMT and tumor metastasis through miRNAs, we primarily determined the miRNA profiles in the EPLC-32 M1 with and without MCRS1 silencing. This research strategy is demonstrated in Figure 5a. In total, 26 known miRNAs and 29 novel miRNAs were differentially expressed after MCRS1 silencing (Additional file 5 and Additional file 6), with miR-155, miR-210, and miR-383 as the most affected. As these 3 miRNAs have been reported to be involved in tumor development and progression [21–23], we selected them for further investigation.

QRT-PCR validation of the three candidate miRNAs demonstrated that miR-155 expression was significantly up-regulated in the NSCLC cell lines (Figure 5b) and NSCLC tissues (Figure 5c). Because MCRS1 is a transcription factor and may target the promoter of the miR-155 host gene (MIR155HG) through bioinformatic prediction (Figure 5d Left), we performed a ChIP to determine whether MCRS1 is targeted to the miR-155 promoter. Indeed, the ChIP results demonstrated that MCRS1 was bound to the promoter region of the miR-155 gene (Figure 5d Right). Moreover, the expression of miR-155 was markedly attenuated in the EPLC-32 M1 and NCI-H292 after MCRS1 silencing (Figure 5e, Additional file 7).

A previous study showed that miR-155 induced EMT by targeting RhoA at the post-transcriptional level [24]. In this study, we found that RhoA is a target of miR-155 in EPLC-32 M1 and NCI-H292, with the expression of RhoA protein being up-regulated after MCRS1 silencing (Figure 5f, 5g). Furthermore, the treatment with miR-155 mimic significantly rescued the cellular proliferation and invasion abilities in MCRS1-depleted cells, whereas miR-155 down-regulation abrogated the effects of MCRS1 in the control cells (Figure 5h, 5i). In clinical samples (6 noncancerous lung tissue, 8 NSCLCs without metastasis, and 7 NSCLCs with metastasis), levels of miR-155 were associated with the status of tumor metastasis (Figure 5j). Summarily, these data suggested that miR-155 could be a downstream node of MCRS1 in the development and progression of NSCLC.

### MCRS1 is directly regulated by miR-129* (miR-129-1-3p) in lung cancer cells

**Figure 6:**
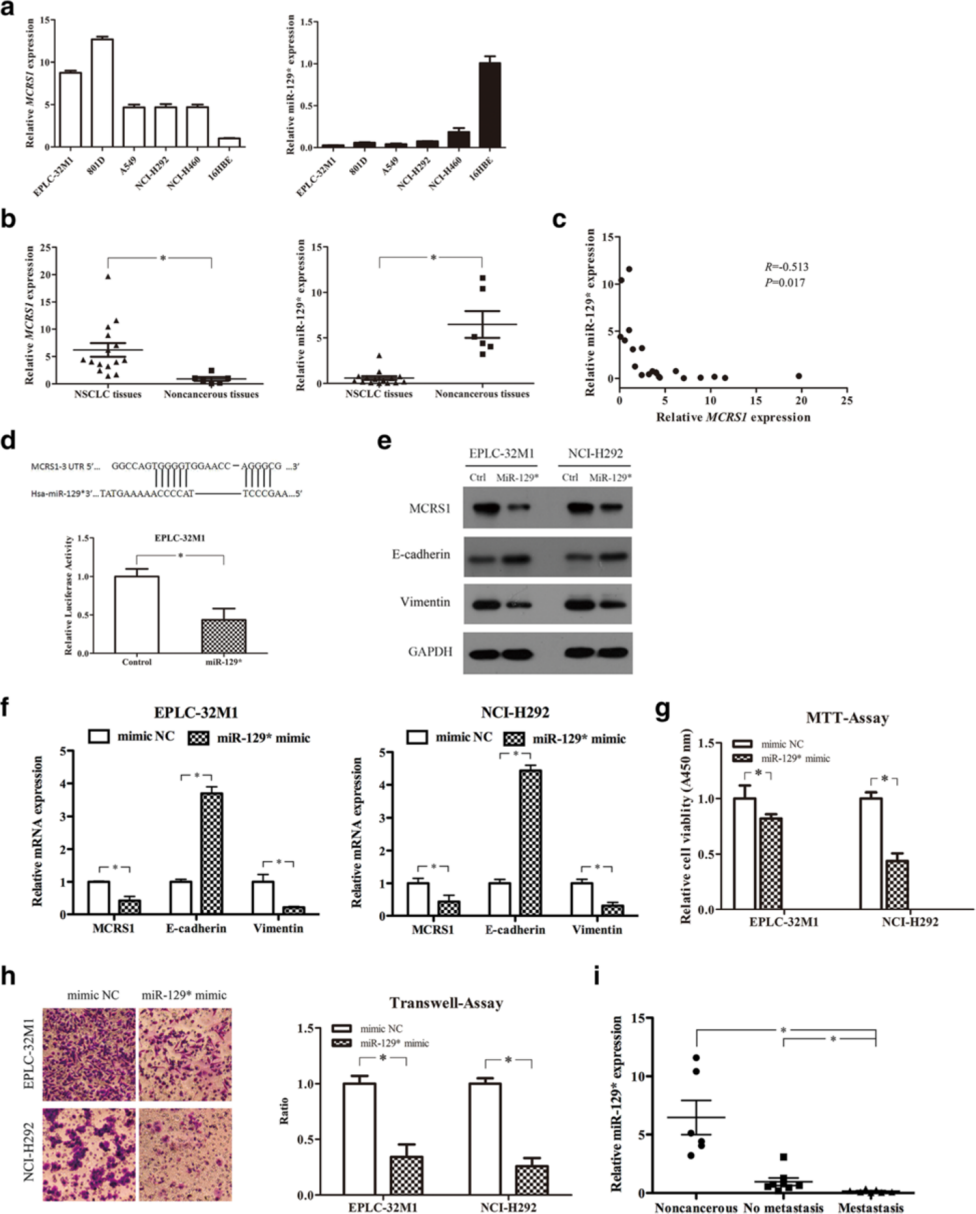
miR-129* directly regulated MCRS1 expression in NSCLC cells. (a) Expression of MCRS1 and miR-129* in NSCLC cell lines. **(b)** The level of miR-129* in clinical samples (Student’s t-test, *P <0.05). **(c)** Correlation analysis of miR-129* and MCRS1 mRNA levels in NSCLC tissues (Pearson’s method, R = -0.513, *P <0.05). **(d)** Top: MCRS1 was predicted as a target of miR-129*. Bottom: Validation of the direct targeting of MCRS1 by miR-129* using a luciferase reporter assay (Student’s t-test, *P <0.05). **(e and f)** Relative mRNA and protein expressions of MCRS1, E-cadherin, and Vimentin in cells transfected with the miR-129* mimic or mimic NC (Student’s t-test, *P <0.05). MTT assays **(g)** and matrigel invasion assays **(h)** of EPLC-32 M1 and NCI-H292 cells transfected with the miR-129* mimic or mimic NC (Student’s t-test, *P <0.05). **(i)** The relationship between the level of miR-129* expression and the status of tumor metastasis (*P <0.05; one-way ANOVA and Student’s t-test). NC: negative control; Noncancerous: noncancerous lung tissues; No metastasis: NSCLCs without metastasis; Metastasis: NSCLCs with metastasis.

Many oncogenes and tumor suppressor genes are regulated by miRNAs [18]. Thus, to identify miRNAs that may regulate MCRS1 expression, miRNA profiles were determined in the NSCLC cell lines (EPLC-32 M1, A549, and 801D) and 16HBE. Compared with 16HBE, in total, 87 consensus miRNAs were differentially expressed in NSCLC cells (Additional file 8). We then used the miRWalk web tool to predict seven miRNAs that target MCRS1 (Additional file 9). The research strategy is described in Additional file 10, and miR-129* and miR-1299 were identified as candidates. We examined the expression of these miRNAs in a series of lung cancer cell lines and found that the expression of miR-129* correlated inversely with MCRS1 expression (Figure 6a). Moreover, we confirmed the data from the cultured cells in the 15 NSCLC tissues and 6 noncancerous lung tissues. As shown in Figure 6b and 6c, miR-129* was remarkably down-regulated in the NSCLC tissues and appeared to be inversely correlated with MCRS1 mRNA expression.

To test whether MCRS1 is a direct target of miR-129*, we performed a luciferase reporter assay by constructing reporter genes containing the MCRS1 3’-UTR and found that the luminescence intensity was significantly reduced following the addition of a miR-129* mimic (Figure 6d). The forced expression of miR-129* significantly suppressed MCRS1 expression at both the mRNA and protein levels (Figure 6e, 6f). Altogether, these data strongly suggest that MCRS1 was directly regulated by miR-129* in the NSCLC cells.

Considering the novel discovery of miR-129* in NSCLC, we assessed the potential effects of miR-129* on EMT process. The forced expression of miRNA-129* significantly induced the up-regulation of E-cadherin (epithelial marker) and down-regulation of Vimentin (mesenchymal marker) at both mRNA and protein levels (Figure 6e, 6f). Moreover, the ectopic expression of miRNA-129* significantly reduced cellular proliferation (Figure 6g) and invasion (Figure 6h). Additionally, miR-129* expressions were analyzed in clinical samples consisting of 6 noncancerous lung tissues, 8 NSCLCs without metastasis, and 7 NSCLCs with metastasis. As shown in Figure 6i, the value of miR-129* expression were related with the status of tumor metastasis.

Summarily, these data demonstrated that miR-129* could be a tumor suppressor that affects cellular behavior by modulating MCRS1 expression.

## Discussion

Tumor invasion and metastasis have been identified as classical hallmarks of cancer malignancy. Metastatic dissemination, disease relapse, and drug resistance are major causes of a poor clinical outcome in cancer patients, and strong evidence has demonstrated that these processes are closely associated with the EMT program [13, 14]. The present study is the first to demonstrate that MCRS1 is a regulator of the EMT program in NSCLC cells. We confirmed this finding by providing the following evidence. 1) EPLC-32 M1, a highly invasive lung cell line, underwent a morphological change from a spindle-like shape to a round shape after MCRS1 silencing. 2) MCRS1 depletion inhibited cellular invasion and reduced monolayer permeability *in vitro.* 3) MCRS1 depletion resulted in the up-regulated expression of ZO-1 and Occludin and E-cadherin, core-constituent molecules of epithelial TJs and adherent junctions (AJs), respectively, which mediate the organization of these junctions. The down-regulation of epithelial junction molecules has been generally accepted as a hallmark of EMT and has also been shown to directly contribute to the invasion of cancer cells [13], and the destruction of TJs can increase epithelial permeability. 4) The metastatic capacity of NSCLC cells was attenuated by MCRS1 depletion *in vivo*, and MCRS1 mRNA expression was associated with tumor metastasis in NSCLC patients. 5) TGF-β treatment simultaneously induced MCRS1 up-regulation and the EMT program in 16HBE. 6) The down-regulation of MCRS1 in NSCLC cells increased the sensitivity of these cells to cisplatin and cetuximab, and decreased the CD44-positive CSC-like cell population. Accumulating evidence has suggested that EMT contributes to the drug resistance and the acquisition of stem cell-like properties [14]. These observations could be considered as additional proofs that MCRS1 overexpression promotes the EMT in NSCLCs. In summary, MCRS1 overexpression contributes to the EMT program in NSCLC cells, and this EMT program may be involved in tumor metastasis. Because metastasis is a very significant factor in the clinical prognosis of patients and MCRS1 overexpression was observed in almost all NSCLC tissues, we speculate that therapies directed at preventing the EMT program mediated by MCRS1 might be a potential treatment for NSCLC, an avenue that is worthy of further study.

To clarify the downstream effectors of MCRS1 in NSCLC, we compared the mRNA expression profiles of cultured cells with or without MCRS1 silencing. The differentially expressed genes were mainly involved in cell cycle, proliferation, apoptosis, EMT process and invasion. The differentially expressed genes which are associated with cell proliferation, included MAPK, P53, Bcl-2, CDK4/6, Cyclin A/B, and so on. In previous study, we found that MCRS1 induced the proliferation of NSCLC [3]. We thought that these differentially expressed genes related to proliferation might be involved in mechanisms by which MCRS1 contributes to the proliferation of NSCLC. Here, we focused on the differentially expressed genes related to EMT process and metastasis. These differentially expressed genes directly related to the EMT program, cellular invasion, and tumor metastasis were grouped into the following families: cell-cell adhesion, cell-extracellular matrix protein (ECM) adhesion, stemness, cellular junctions, and Notch signaling. Surprisingly, the classical EMT transcription factors (Snail, Twist, and Slug) were not found to be differentially expressed. Several molecules related to EMT are discussed below. 1) Occludin and ZO-1 are a transmembrane protein and a cytoplasmic plaque protein at TJs, respectively, and both play important roles in maintaining the TJ structure and function. TJs contribute to cell-cell attachment and maintain epithelial cell integrity and polarity [25], and the destruction of TJs can increase epithelial permeability and cellular invasion, promoting tumor metastasis [26]. In our study, MCRS1 overexpression increased epithelial permeability and reduced both ZO-1 and Occludin expression (Figure 1d, 1e), indicating that MCRS1 overexpression may abolish the functions of TJs and promote cellular invasion and tumor metastasis through the suppression of TJ molecules. 2) E-cadherin is the major transmembrane protein of AJs and mediates Ca^2+^-dependent cell-cell adhesion; the loss of E-cadherin results in AJ disruption and facilitates tumor invasion and metastasis [27]. In our study, E-cadherin was found to be up-regulated at both the mRNA and protein levels after MCRS1 knockdown, suggesting that MCRS1 overexpression can lead to AJ disruption, cellular invasion, and tumor metastasis. 3) DSG2 is a transmembrane protein found in desmosomes and plays an important role in cell adhesion [28], the loss of which facilitates the EMT program and tumor metastasis [29]. We found that MCRS1 overexpression suppressed the expression of DSG2 mRNA, indicating that MCRS1 overexpression could impair cell adhesion and increase tumor invasion and metastasis. However, promoters of ZO-1, Occludin, E-cadherin and DSG2 genes were not directly recognized by a transcriptional complex containing MCRS1. The mechanisms by which MCRS1 regulates these genes remain unresolved. We noted that several molecules related to the EMT program were also differentially expressed after MCRS1 silencing, including ITGA6 and Notch3. The former is a marker of stems cells that can enhance and maintain the stemness of cancer cells [30]. The latter is a member of the Notch family of transmembrane receptors and can induce or suppress the EMT program depending on the cancer type; Notch3 knockdown was reported to result in the reduction of lung CSCs [31]. Altogether, these results indicated that MCRS1 overexpression can influence multiple molecules and pathways and promote the EMT program, cellular invasion, and tumor metastasis.

Emerging evidence has demonstrated that miRNAs play vital roles in the regulation of various biological and pathological processes, including the EMT program, cell invasion, and tumor metastasis [18, 20]. By assessing miRNA expression profiles after MCRS1 depletion, we found that miR-155 is a downstream effector of MCRS1. Moreover, miR-155 was up-regulated in NSCLC tissues and cultured cells, and its overexpression was correlated with the MCRS1 expression and the status of tumor metastasis. ChIP assay demonstrated that MCRS1 binds to the miR-155 promoter. Our interference experiments also confirmed that miR-155 expression could be induced by MCRS1 over-expression. The forced expression of miR-155 recapitulated the effect of MCRS1 on cell growth and invasion. MiR-155, which is known as an oncogenic miRNA, is up-regulated in multiple human tumors and plays functional roles in several aspects of tumor development and progression [21, 24, 32]. In our study, the down-regulation of miR-155 was found to reduce cellular growth and invasion, and *vice versa*. However, the down-regulation of miRNA-155 did not alter the expression of ZO-1 and Occludin but did change the levels of E-cadherin. Therefore, the down-regulation of the junction molecules induced by MCRS1 was independent of miRNA-155. Recent studies have shown that miR-155 mediates TGF-β-induced EMT by targeting RhoA [24] and induces breast cancer EMT through the loss of C/EBPβ [33]. In the present work, the expression levels of RhoA and E-cadherin proteins were down-regulated after the miRNA-155 mimetic treatment but were up-regulated after MCRS1 silencing. Therefore, we hypothesize that miRNA-155 and RhoA may be functional downstream mediators of the EMT program mediated by MCRS1 in NSCLC cells. These findings provide an additional molecular mechanism of the pathophysiological functions of MCRS1.

Previous studies have demonstrated that many oncogenes are potentially regulated by tumor suppressor miRNAs [18]. To identify whether MCRS1 overexpression is regulated by miRNAs, the miRNA profiles of cultured NSCLC cells and immortalized human bronchial epithelial cells were compared, and the differentially expressed miRNAs were further analyzed using bioinformatic methods to screen the candidates. Among the 7 selected candidates, miR-129* expression was significantly down-regulated and negatively correlated with MCRS1 expression in the NSCLC tissues and cultured cells. The value of miR-129* was inversely correlated with the status of tumor metastasis. Furthermore, MCRS1 expression was regulated by the binding of miR-129* to the 3’-UTR *in vitro*. Collectively, these results suggest that miR-129* expression can suppress oncogenic MCRS1 expression in NSCLC cells. MiR-129* originates from miR-129-1 at the 7q32.1 locus, which is a site commonly deleted in many cancers [34]; indeed, a recent study indicated that the expression of miR-129* is down-regulated in gastric cancer [35]. In our functional test, miR-129* overexpression suppressed the cellular growth and invasion in NSCLC cells. Based on these data, miR-129* could be a tumor suppressor miRNA in NSCLC cells.

## Conclusions

**Figure 7:**
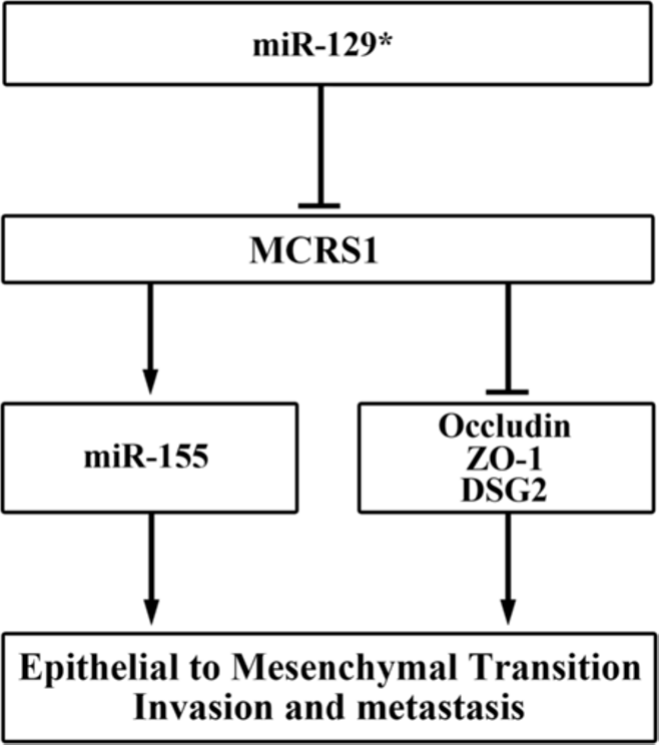
**Proposed model of MCRS1 regulation and its functional roles.**

MCRS1 overexpression promoted the EMT program and tumor invasion/metastasis in NSCLC cells through the up-regulation of miR-155 and the down-regulation of cell junction molecules. Additionally, MCRS1 activation was mediated by miR-129* down-regulation. The miR-129*/MCRS1/miR-155 axis provides a new avenue to understand the mechanism of the tumor invasion and metastasis (Figure 7).

## Methods

### Human tissue specimens and cell lines

Lung cancer tissues were obtained from 35 patients undergoing resection. No patients were treated before undergoing surgery. The tumor samples were from primary tumors. The metastatic status of patients was recorded on the basis of the pathological and clinical examinations at the time of resection. All of these samples were diagnosed according to World Health Organization’s classification and staged based on the International Union Against Cancer [36, 37]. In total, 8 lung tissues, obtained from patients with lung bullous and inflammatory pseudotumor through surgical operation, are used as “noncancerous tissues”. Our study was approved by the local research ethics committee.

Details of all cell lines were provided in Additional file 11.

After 24 hours (h) serum starvation, 16HBE (an immortalized human bronchial epithelial cell line) was treated with TGF-β1 (Invitrogen, Carlsbad, CA, USA) at 15 ng/ml and cultured for 72 h. The transient MCRS1 interference was also carried out in TGF-β1 treated cells as described previously [3].

### RNA interference

The target sequence (GCTGAAGAACAACGGTGAT) was used for MCRS1 interference as described previously [3]. Luciferase shRNA oligonucleotide served as a negative control. The complementary strands of the oligonuleotides were annealed and ligated into prelinearized RNAi-Ready pSIREN-RetroQ vector (Clontech, Palo Alto, CA, USA). Recombinant vectors were then cloned and transfected into PT-67 cells using Lipofectamine 2000 (Invitrogen). After 48 h transfection, the retrovirus–containing medium was filtered and used to infect NSCLC cell lines in the presence of 10ug/ml polybrene (Sigma, St. Louis, MO, USA). After 48 h infection, the cells were selected by puromycin selection (Sigma). Stable subcell-lines with MCRS1 knockdown and control cells were established to further investigate.

### QRT–PCR

For mRNA expression assay, total RNA was isolated from cells using the RNA-Pure kit (QIAGEN, Hilden, Germany). The cDNA was synthesized by M-MLV Reverse Transcriptase (Promega, Madison, WI, USA) using random primer and amplified with specific primers on the StepOne Realtime PCR System (Applied Biosystems, Foster City, CA, USA).

For miRNA expression assay, total RNA was polyadenylated and reverse-transcribed into cDNA using the miScript II RT kit (QIAGEN), and the qRT-PCR analysis was performed using the miScript SYBR Green PCR kit (QIAGEN) according to the manufacturer’s instructions. All primers in our research were listed in Additional file 12.

### Western blot analysis

Cells were harvested and lysed in RIPA buffer (Beyotime Biotechnology, Shanghai, China). Cell lysates were resolved by 8-12% sodium dodecylsulfate polyacrylamide gel electrophoresis (SDS-PAGE) and transferred onto PVDF membranes (Millipore, Bedford, MA, USA). The membrane was incubated with the specific antibodies and all antibodies in our research were listed in Additional file 13.

### Immunofluorescence staining and confocal microscopy

Cells grown on the glass coverslip were fixed in 3.7% formaldehyde solution, permeated with 0.1% Triton X-100, blocked with 1% bovine serum albumin (BSA) in phosphate buffer saline (PBS), and incubated respectively with rhodamine-conjugated phalloidin (Invitrogen) and DAPI (Beyotime Biotechnology) to stain F-actin and nuclei. Then cells were mounted with 50% glycerol and analyzed by confocal laser scanning microscopy (Zeiss LSM510 Meta, Germany).

### Flow cytometric analysis

Expression of cancer stem-like cells markers was evaluated by fluorescence activated cell sorting (FACS). Cells were washed with 2% FBS in PBS and stained with FITC-conjugated anti-CD44 antibody (BD Pharmingen, San Diego, CA, USA). After the staining, cells were washed again, resuspended and analyzed on a FACScan flow cytometer (BD Biosciences, San Jose, CA, USA).

### Cell monolayer integrity assessment

Cell monolayer integrity was assessed by measuring the permeation of horseradish peroxidase (HRP, Sigma) using the Boyden Chamber as our previously described [19]. Briefly, cells were cultured onto the upper chamber membrane with 8 μm pore (BD Bioscience) to complete confluence. Then the medium in the lower and top chamber was replaced with the fresh medium and medium containing HRP, respectively. The medium in the basal chamber was harvested every 5 minutes (min) and transferred into 96-well plates. Then substrate solution (50 mM sodium phosphate buffer, pH 5.0, 342 μM o-dianisidine dihydrochloride, 0.003% H2O2) was added, followed by addition of 0.1% sodium azide. Optical density was read on a microplatereader at 450 nm.

### Cell invasion assay

Cell invasion were performed using 24 transwell chamber with matrigel-coated membrane (pore size = 8 μm; BD Bioscience). 1 × 10^5^ prepared cells were seeded in the upper chamber and incubated for 18-24 h. After the noninvasive cells were removed by a cotton swab, the invasive cells were fixed in 4% paraformaldehyde and stained with crystal violet. The stained cells were viewed and counted using inverted microscope (Nikon, Tokyo, Japan).

### Cell proliferation assay

Cell proliferation assay was based on 3-(4,5-dimethylthiazol-2-yl)-2, 5-Diphenyltetrazolium bromide (MTT) methods. Cells were seeded at a density of 4 × 10^3^ cells /well in 96-well plates for 24 h. For cell growth assay, MTT was added into each well. After the MTT incubation, the medium was removed and followed by addition of Dimethyl sulfoxide (DMSO). The absorbance was read at 570 nm (630 nm as a reference) on a microplate reader (model 680, Bio-Rad Laboratories).

For drug resistance assay, cells were maintained in culture medium containing cisplatin (15 μM) or cetuximab (5 μg/ml) for 72 h, and subsequently analyzed by cell proliferation assay.

### *In vivo*metastasis assay

Female BALB/c nude mice (4 weeks) were purchased from Vital River Laboratories (Beijing, China) and housed under standard condition. The animals were divided into two groups of 6 mice each. The mice of the two groups were implanted intravenously with MCRS1 knockdown cells and control cells (1 × 10^6^ in 0.2 ml PBS) into the lateral tail vein, respectively. The tumors were excised, counted and histopathologically examined by hematoxylin-eosin staining after four weeks. All experiments were performed in accordance with the regulation experimentation for animal and approved by the responsible authorities.

### mRNA/miRNA expression profiling

Total RNA was isolated using the Trizol reagent (Sigma) following the manufacturer’s protocol. Determination of mRNA profiling was performed in EPLC-32 M1 cells with and without MCRS1 knockdown using Agilent 60 K Human Gene Expression array by CapitalBio Corporation (Beijing, China; http://www.capitalbio.com). The differentially expressed genes were subjected to Gene Ontology (GO) and Kyoto Encyclopedia of Genes and Genomes (KEGG) pathway analyses using the Mas 3.0 molecule annotation system (http://bioinfo.capitalbio.com/mas3).

MiRNA expression profiling was determined in A549, 801D, and 16HBE, as well as EPLC-32 M1 cells with and without MCRS1 knockdown using Hiseq 2000 platform by BGI Tech (Shenzhen, China; http://bgitechsolutions.com). The miRWalk database (http://www.umm.uni-heidelberg.de/apps/zmf/mirwalk/micrornapredictedtarget.html) was used to predict the target genes of the miRNAs [38].

### Transient transfection of miRNA mimics or inhibiters

MiRNA mimics, 2’-O-methylated miRNA antisense oligonucleotides (ASO), and their cognate controls were purchased from RiBoBio Company (Guangzhou, China). The transfection was performed using Lipofectamine 2000 (Invitrogen) following the manufacturer’s instructions. For the transfection, 100 nmol/L miRNA mimic and 150 nmol/L ASO were used.

### ChIP assay

ChIP assays were performed using the EZ-Magna ChIP kit (2165871; Millipore, Merck KGaA, Darmstadt, Germany) according to manufacturer’s instructions. Briefly, 3 × 10^7^ cells were collected and cross-linked with 1% formaldehyde at room temperature for 10 min and then lysed in cell lysis buffer containing protease inhibitor cocktail II. After centrifugation, the cell pellet were resuspended in nuclear lysis buffer containing cocktail II and sonicated on ice to an average length of approximately 250 bp with a Bioruptor sonicator. The sonicated chromatin was then immunoprecipitated using an anti-MCRS1 antibody (Additional file 13) or normal mouse IgG (the negative control) and protein G magnetic beads overnight at 4°C with rotation. After removing the supernatant using a magnetic separator (Invitrogen), the protein/DNA complexes were washed and eluted, and the crosslinks were reversed at 62°C. The input and immunoprecipitated DNAs were purified using Spin columns and then analyzed by PCR.

### MCRS1 3’-UTR construction and luciferase reporter assay

To obtain the luciferase construct, the full-length 3’-UTR of MCRS1 (containing the binding sites for miR-129*) was amplified from the genomic DNA of EPLC-32 M1 cells and was inserted between the MluI and BgIII restriction sites in the pGL-3 basic vector (Promega).

For the luciferase assays, a mixture of the pGL-3’-UTR vector and Renilla luciferase plasmid (pRL-TK) was cotransfected into the cells with the miR-129* mimic or its negative control using Lipofectamine 2000. The pRL-TK plasmid was used as an internal control. After transfection, the luciferase activities were measured using Dual-Luciferase Reporter System (Promega) according to the manufacturer’s instructions.

### Statistical analysis

All the data are presented as the mean ± standard deviation (SD) from at least three independent experiments. The data were analyzed using SPSS 17.0 software package (Chicago, IL, USA). The measurement data (levels of mRNA and miRNA, HRP permeability, cell invasion and proliferation) were analyzed by Student’s t-test or a one-way ANOVA, as appropriate. The relationship between the miRNA levels and gene expression was determined using Pearson’s correlation coefficient test. The level of statistical significance was set at 0.05 for all the tests.

## Electronic supplementary material

Additional file 1: **Expression of MCRS1 by western blotting and** **qRT-PCR.** **(a)** Results of MCRS1 protein in seven NSCLC cell lines and an immortalized human bronchial epithelial cell line (16HBE). **(b)** Results of MCRS1 protein in EPLC-32 M1 and NCI-H292 cells with (Msh3) and without (Luc) MCRS1 knockdown. **(c)** Results of MCRS1 mRNA in EPLC-32 M1 and NCI-H292 cells with (Msh3) and without (Luc) MCRS1 knockdown. (TIFF 773 KB)

Additional file 2: **Top 10 alterations in cellular functions related to differentially expressed genes after MCRS1 silencing in EPLC-32 M1 cells using GO term analysis.** (TIFF 13 MB)

Additional file 3: **Differentially expressed genes related to the EMT program based on the microarray data of EPLC-32 M1 and MCRS1-depleted EPLC-32 M1 cells using KEGG analysis.** (DOC 88 KB)

Additional file 4: **Studying relationships between ZO-1, Occludin, E-cadherin, and DSG2 genes as well as MCRS1 through bioinformatics analyses and ChIP-PCR assay.** (DOC 46 KB)

Additional file 5: **Differentially expressed miRNAs in EPLC-32 M1 and MCRS1-depleted EPLC-32 M1 cells: known miRNAs.** (DOC 46 KB)

Additional file 6: **Differentially expressed miRNAs in EPLC-32 M1 and MCRS1-depleted EPLC-32 M1 cells: novel miRNAs.** (DOC 50 KB)

Additional file 7: **Potential miRNAs downstream of MCRS1 were examined by qRT-PCR.** Expression of miR-210 **(a)** and miR-383 **(b)** in EPLC-32 M1 and NCI-H292 cells with (Msh3) and without (Luc) MCRS1 knockdown. (Student’s t-test, *P <0.05). (TIFF 389 KB)

Additional file 8: **Heatmap of the differentially expressed miRNAs in three NSCLC cell lines (A549, 801D, and EPLC-32 M1) compared to the immortalized human bronchial epithelial cell line (16HBE).** Green, down-regulation; red, up-regulation. (TIFF 1 MB)

Additional file 9: **The seven differentially expressed miRNAs selected through the integrated analysis of miRNA expression profiles with miRNA target prediction.** (DOC 37 KB)

Additional file 10: Schematic diagram illustrating the research strategy focusing on miR-129*. This study was initially performed to identify differential miRNAs using the miRNA-sequence method; 7 miRNAs targeting MCRS1 were predicted using bioinformatics. miR-129* and miR-1299 were subsequently chosen for further validation because of the inverse relationship between these two miRNAs and MCRS1 expression. qRT-PCR assays confirmed that the expression of miR-129* was significantly down-regulated in NSCLC cell lines. (TIFF 483 KB)

Additional file 11: **The cell lines used in this study.** (DOC 34 KB)

Additional file 12: **The primers used in this study.** (DOC 41 KB)

Additional file 13: **The antibodies used in this study.** (DOC 42 KB)

## Abbreviations

MCRS1: Microspherule protein 1
EMT: The epithelial-mesenchymal transition
NSCLC: Non-small cell lung cancer
miRNA: MicroRNA
TJs: Tight junctions
AJs: Adherent junctions
DSG2: Desmoglein 2
HRP: Horseradish peroxidase
CSC: Cancer stem cell
qRT–PCR: Quantitative real-time polymerase chain reaction
ChIP: Chromatin immunoprecipitation
GO term: Gene ontology terms
KEGG analysis: Kyoto encyclopedia of genes and genomes analysis.
