# Supplementary figures and images for: MCRS1 overexpression, which is specifically inhibited by miR-129*, promotes the epithelial-mesenchymal transition and metastasis in non-small cell lung cancer
Source: Mol Cancer. 2014 Nov 6;13:245. doi: 10.1186/1476-4598-13-245 (PMC4233086; doi:10.1186/1476-4598-13-245)

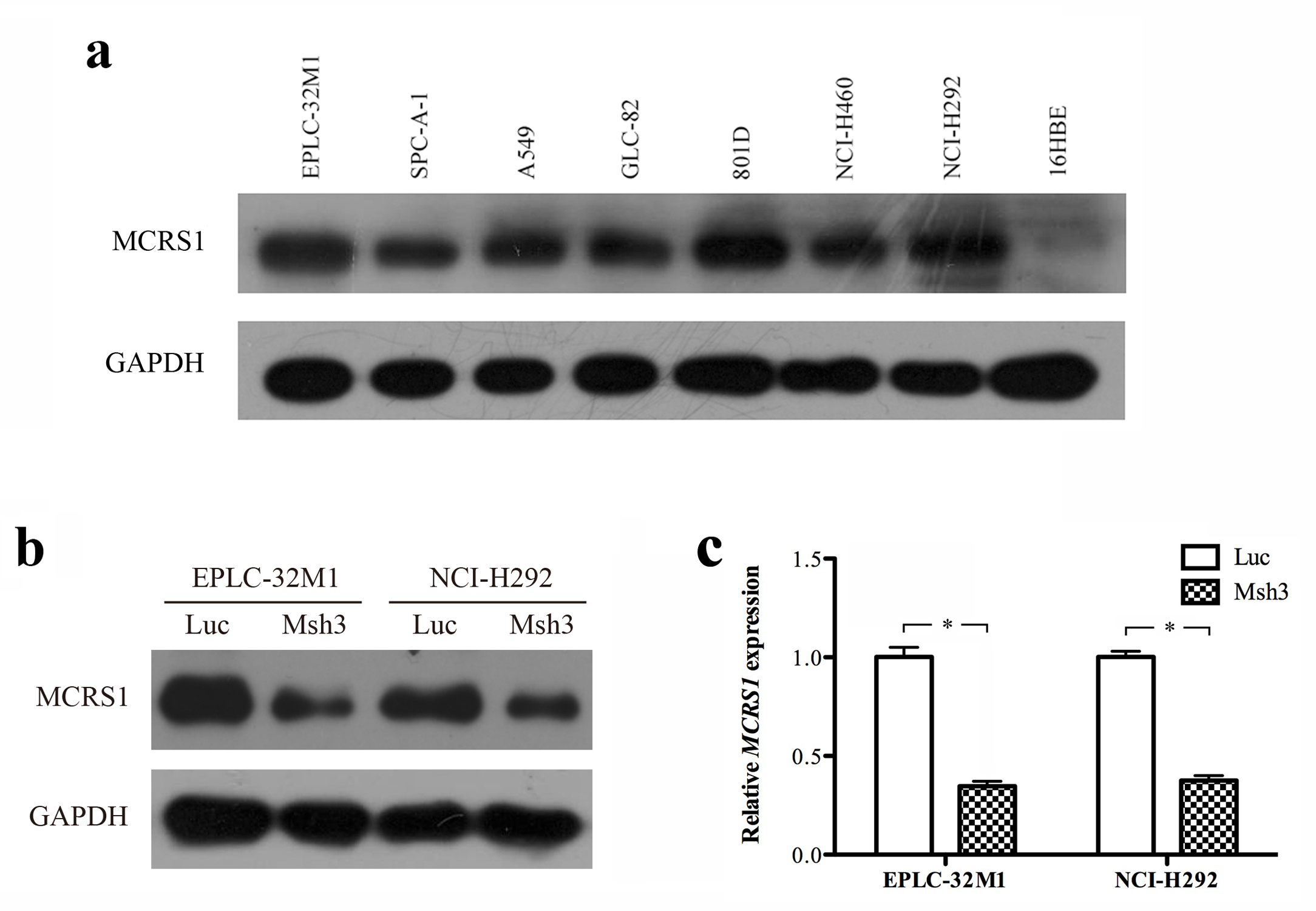

Supplement: Supplementary file 1 — Additional file 1: Expression of MCRS1 by western blotting and qRT-PCR. (a) Results of MCRS1 protein in seven NSCLC cell lines and an immortalized human bronchial epithelial cell line (16HBE). (b) Results of MCRS1 protein in EPLC-32 M1 and NCI-H292 cells with (Msh3) and without (Luc) MCRS1 knockdown. (c) Results of MCRS1 mRNA in EPLC-32 M1 and NCI-H292 cells with (Msh3) and without (Luc) MCRS1 knockdown. (TIFF 773 KB) [file 12943_2014_1444_MOESM1_ESM.tiff]

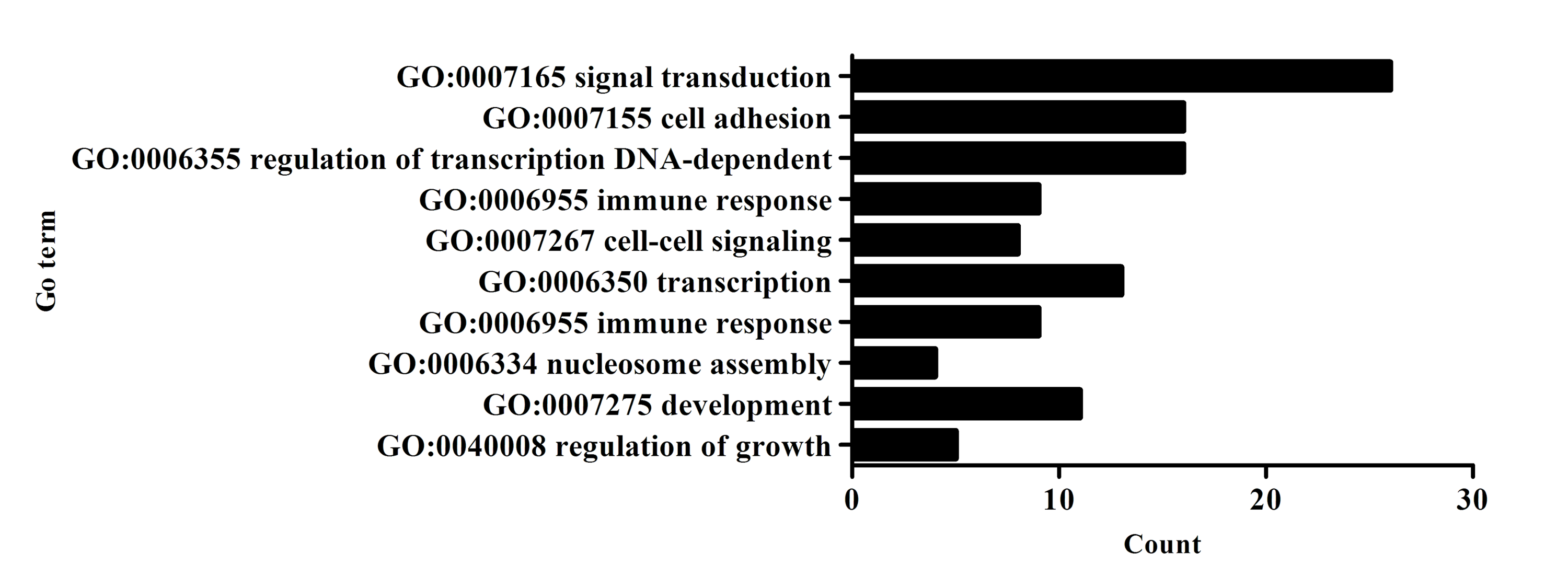

Supplement: Supplementary file 2 — Additional file 2: Top 10 alterations in cellular functions related to differentially expressed genes after MCRS1 silencing in EPLC-32 M1 cells using GO term analysis. (TIFF 13 MB) [file 12943_2014_1444_MOESM2_ESM.tiff]

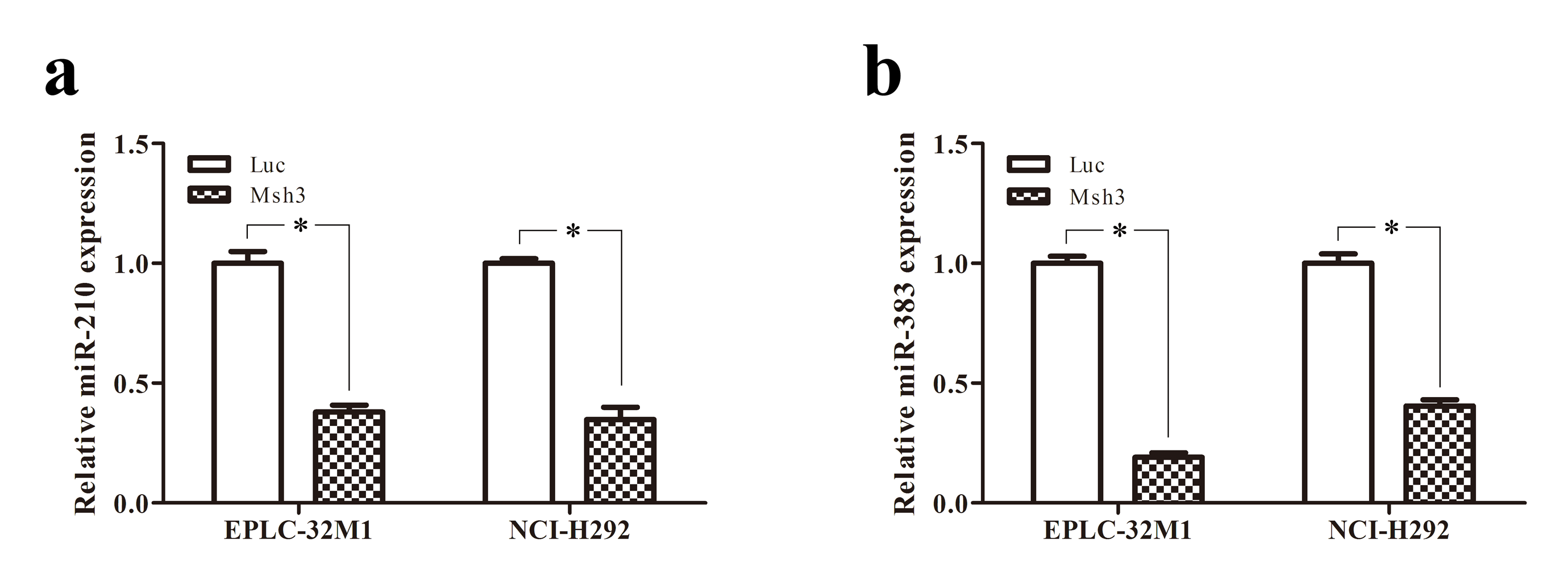

Supplement: Supplementary file 7 — Additional file 7: Potential miRNAs downstream of MCRS1 were examined by qRT-PCR. Expression of miR-210 (a) and miR-383 (b) in EPLC-32 M1 and NCI-H292 cells with (Msh3) and without (Luc) MCRS1 knockdown. (Student’s t-test, *P <0.05). (TIFF 389 KB) [file 12943_2014_1444_MOESM7_ESM.tiff]

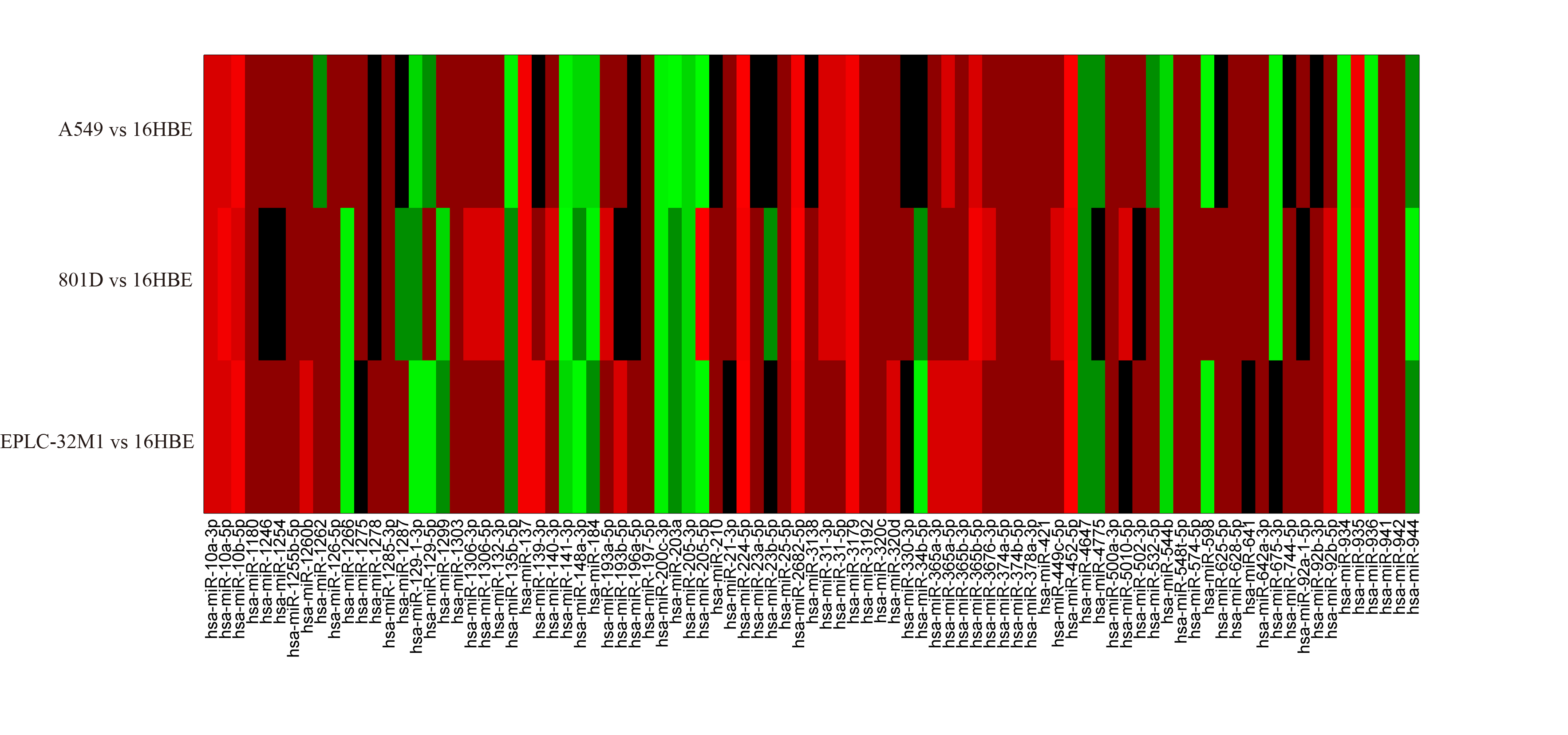

Supplement: Supplementary file 8 — Additional file 8: Heatmap of the differentially expressed miRNAs in three NSCLC cell lines (A549, 801D, and EPLC-32 M1) compared to the immortalized human bronchial epithelial cell line (16HBE). Green, down-regulation; red, up-regulation. (TIFF 1 MB) [file 12943_2014_1444_MOESM8_ESM.tiff]

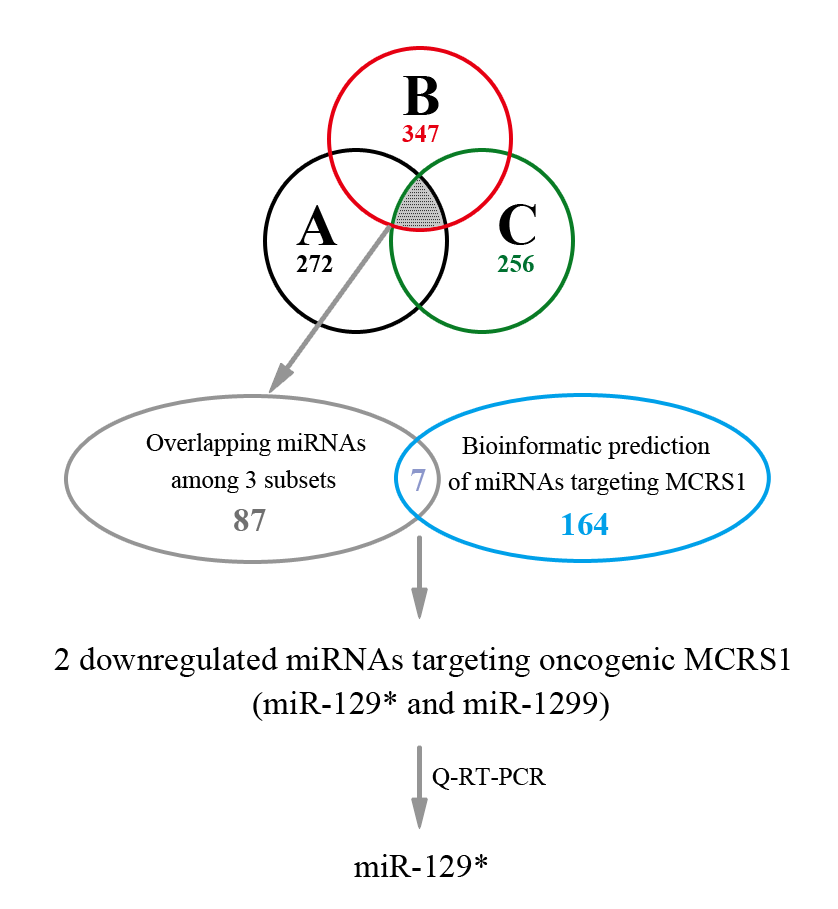

Supplement: Supplementary file 10 — Additional file 10: Schematic diagram illustrating the research strategy focusing on miR-129*. This study was initially performed to identify differential miRNAs using the miRNA-sequence method; 7 miRNAs targeting MCRS1 were predicted using bioinformatics. miR-129* and miR-1299 were subsequently chosen for further validation because of the inverse relationship between these two miRNAs and MCRS1 expression. qRT-PCR assays confirmed that the expression of miR-129* was significantly down-regulated in NSCLC cell lines. (TIFF 483 KB) [file 12943_2014_1444_MOESM10_ESM.tiff]
