# Supplementary material for: MCRS1 overexpression, which is specifically inhibited by miR-129*, promotes the epithelial-mesenchymal transition and metastasis in non-small cell lung cancer
Source: Mol Cancer. 2014 Nov 6;13:245. doi: 10.1186/1476-4598-13-245 (PMC4233086; doi:10.1186/1476-4598-13-245)
Supplement: Supplementary file 3 — Additional file 3: Differentially expressed genes related to the EMT program based on the microarray data of EPLC-32 M1 and MCRS1-depleted EPLC-32 M1 cells using KEGG analysis. (DOC 88 KB) [file 12943_2014_1444_MOESM3_ESM.doc]

**Additional file 3: Differentially expressed genes related to the EMT program based on the microarray data of EPLC-32M1 and MCRS1-depleted EPLC-32M1 cells using KEGG analysis.**

|  |  | Genbank | Gene tittle | Fold change | P-value |  | | |
| --- | --- | --- | --- | --- | --- | --- | --- | --- |
| **Regulation of actin cytoskeleton** | | | |  |  |  | | |
| ITGA6 |  | NM_000210 | integrin, alpha 6 | -2.3408 | * |  | | |
| BDKRB2 |  | NM_000623 | bradykinin receptor B2 | 2.3148 | * |  | | |
| CHRM1 |  | NM_000738 | cholinergic receptor, muscarinic 1 | -2.8064 | * |  | | |
| APC |  | NM_001127511 | adenomatous polyposis coli | 2.171 | * |  | | |
| EGF |  | NM_001963 | epidermal growth factor (EGF), transcript variant 1 | -2.015 | * |  | | |
| FGF12 |  | NM_004113 | fibroblast growth factor 12 (FGF12), transcript variant 2 | 5.035 | * |  | | |
| FGF20 |  | NM_019851 | fibroblast growth factor 20 (FGF20) | -2.0587 | * |  | | |
| FGF14 |  | NM_175929 | fibroblast growth factor 14 (FGF14) | 2.086 | * |  | | |
| **Cytokine-cytokine receptor interaction** | | | |  |  |  | | |
| IL11 |  | NM_000641 | interleukin 11 | 9.7943 | * |  | | |
| EGF |  | NM_001963 | epidermal growth factor (EGF), transcript variant 1 | -2.015 | * |  | | |
| IL7R |  | NM_002185 | interleukin 7 receptor (IL7R) | 2.266 | * |  | | |
| CCL2 |  | NM_002982 | chemokine (C-C motif) ligand 2 | -2.0974 | * |  | | |
| TNFSF4 |  | NM_003326 | tumor necrosis factor (ligand) superfamily, member 4 | 2.337 | * |  | | |
| CCL20 |  | NM_004591 | chemokine (C-C motif) ligand 20 | 2.301 | * |  | | |
| IL28RA |  | NM_170743 | interleukin 28 receptor, alpha (interferon, lambda receptor) | 2.087 | * |  | | |
| TPO |  | NM_175719 | thyroid peroxidase (TPO), transcript variant 2 | 2.372 | * |  | | |
| **Focal adhesion** | | | |  |  |  |  | * |
| AKT3 |  | ENST00000366539 | v-akt murine thymoma viral oncogene homolog 3 (protein kinase B, gamma) | 2.610 | * |  | | |
| ITGA6 |  | NM_000210 | integrin, alpha 6 | -2.3408 | * |  | | |
| COL6A1 |  | NM_001848 | collagen, type VI, alpha 1 (COL6A1) | -2.6568 | * |  | | |
| EGF |  | NM_001963 | epidermal growth factor (EGF), transcript variant 1 | -2.015 | * |  | | |
| LAMA1 |  | NM_005559 | laminin, alpha 1 (LAMA1) | 2.149 | * |  | | |
| **Cell adhesion Molecules (CAMs)** | | | |  |  |  |  | * |
| ITGA6 |  | NM_000210 | integrin, alpha 6 | -2.3408 | * |  | | |
| PECAM1 |  | NM_000442 | platelet/endothelial cell adhesion molecule (PECAM1) | 2.055 | * |  | | |
| HLA-DMB |  | NM_002118 | major histocompatibility complex, class II, DM beta (HLA-DMB) | 2.655 | * |  | | |
| CDH1 |  | NM_004360 | cadherin 1, type 1,E-cadherin (epithelial) | 2.130 | * |  | | |
| **Tight junction** | | | |  |  |  |  | * |
| TJP1 |  | NM_003257 | tight junction protein 1 (zona occludens 1) | 2.650 | * |  | | |
| OCLN |  | NM_002538 | occludin | 2.553 | * |  | | |
| AKT3 |  | ENST00000366539 | v-akt murine thymoma viral oncogene homolog 3 (protein kinase B, gamma) | 2.610 | * |  | | |
| MLLT4 |  | NM_001040000 | myeloid/lymphoid or mixed-lineage leukemia | 2.187 | * |  | | |
| PRKCZ |  | NM_002744 | protein kinase C, zeta (PRKCZ), transcript variant 1 | 2.088 | * |  | | |
| RAB3B |  | NM_002867 | member RAS oncogene family (RAB3B) | 2.045 | * |  | | |
| **ECM-receptor interaction** | | | |  |  |  | | |
| ITGA6 |  | NM_000210 | integrin, alpha 6 | -2.3408 | * |  | | |
| COL6A1 |  | NM_001848 | collagen, type VI, alpha 1 (COL6A1) | -2.6568 | * |  | | |
| LAMA1 |  | NM_005559 | laminin, alpha 1 (LAMA1) | 2.149 | * |  | | |
| **Notch signaling pathway** | | | |  |  |  |  | * |
| NOTCH3 |  | NM_000435 | notch 3 | -2.601 | * |  | | |
| HES1 |  | NM_005524 | hairy and enhancer of split 1 | 2.465 | * |  | | |
| **Adherent junction** | | | |  |  |  |  | * |
| MLLT4 |  | NM_001040000 | myeloid/lymphoid or mixed-lineage leukemia | 2.186 | * |  | | |
| CDH1 |  | NM_004360 | cadherin 1, type 1,E-cadherin (epithelial) | 2.130 | * |  | | |
|  |  |  |  |  |  |  | | |

-: downregulation; +: upregulation; *P<0.05
