# Supplementary material for: MCRS1 overexpression, which is specifically inhibited by miR-129*, promotes the epithelial-mesenchymal transition and metastasis in non-small cell lung cancer
Source: Mol Cancer. 2014 Nov 6;13:245. doi: 10.1186/1476-4598-13-245 (PMC4233086; doi:10.1186/1476-4598-13-245)
Supplement: Supplementary file 4 — Additional file 4: Studying relationships between ZO-1, Occludin, E-cadherin, and DSG2 genes as well as MCRS1 through bioinformatics analyses and ChIP-PCR assay. (DOC 46 KB) [file 12943_2014_1444_MOESM4_ESM.doc]

**Additional file 4: Studying relationships between ZO-1, Occludin, E-cadherin, and DSG2 genes as well as MCRS1 through bioinformatics analyses and ChIP-PCR assay.**

UniProt website ([http://www.uniprot.org](http://www.uniprot.org/)) and TFBID software (<http://tfbind.hgc.jp/>) were respectively employed to search for MCRS1 complexes and to predict binding sites of MCRS1 complexes. MCRS1 complexes did not bind to the promoters of ZO-1, Occludin, E-cadherin or DSG2 genes.

The promoter regions of ZO-1, Occludin, E-cadherin or DSG2 were determined via Transcriptional Regulatory Element Database (TRED) (<https://cb.utdallas.edu/cgi-bin/TRED/tred.cgi?process=home>). Then we designed the paired primers and performed ChIP-PCR assay to investigate whether MCRS1 could bind to these promoter regions. The primers and results were demonstrated in the following:

| **Gene** | **Forward primers (5'-3');**  **Reverse primers (5'-3')** | **Results of ChIP-PCR** |
| --- | --- | --- |
| **ZO-1**  **(TJP1)** | 1. GGTCTAATGTGGGGTGTGGG;   AGAAGCGTTGCTCTCGTTCA | Negative |
|  | 1. CTGTCTAGATGGGCGGGAAA;   GCAGCCTGAGAAACACCCTA | Negative |
|  | 1. TAGGGTGTTTCTCAGGCTGCTG;   CATTTTCTGCAAGGCGCGTCA | Negative |
| **Occludin (OCLN)** | 1. AAGCACTACAGGTTGGTAAACA;   TTTACACATCACCACCCCAGG | Negative |
|  | 1. GGAACTAAGGTGCTTTTTCGTTT;   TGGCTCTTTGGCCTGAGAAA | Negative |
|  | 1. CACACCACACCTACACTCCC;   GAGTCCCACCTGCTGCG | Negative |
|  | 1. CTCCCTCCCTGCTTCCTCT;   ATGCGCACCAACGTGGAA | Negative |
| **E-cadherin (CDH1)** | 1. GACTTGCGAGGGACGCATTC;   CTCCTCAGGACCCGAACTTT | Negative |
|  | 1. CGGATCCCCTGACTTGCGA;   CTTTCTTGGAAGAAGGGAAGCG | Negative |
|  | 1. TTCCCATTAGGAGGGTGGAGA;   TTTTAACTGTAGAGCTTCATGGGT | Negative |
| **Desmoglein 2**  **(DSG2)** | 1. GGAAGTTTCGTAATCCGGTGC;   ACAACGTGATTGCGAGGTGA | Negative |
|  | 1. AGGCTCGTCCATTACTTTCAGT;   AACGTGATTGCGAGGTGAGA | Negative |
|  | 1. GTCAATTTAACATCTCACCTCGCA;   CAACGCACGGGGAAGTTTTG | Negative |
