# Supplementary material for: MCRS1 overexpression, which is specifically inhibited by miR-129*, promotes the epithelial-mesenchymal transition and metastasis in non-small cell lung cancer
Source: Mol Cancer. 2014 Nov 6;13:245. doi: 10.1186/1476-4598-13-245 (PMC4233086; doi:10.1186/1476-4598-13-245)
Supplement: Supplementary file 5 — Additional file 5: Differentially expressed miRNAs in EPLC-32 M1 and MCRS1-depleted EPLC-32 M1 cells: known miRNAs. (DOC 46 KB) [file 12943_2014_1444_MOESM5_ESM.doc]

**Supplementary Table 5: Differentially expressed miRNAs in EPLC-32M1 and MCRS1-depleted EPLC-32M1 cells: known miRNAs.**

| **Name** | **Fold change**  **Log2>=1** | **Sig-lable of P value** |
| --- | --- | --- |
| **Up-regulated miRNAs** |  |  |
| hsa-miR-4488 | 1.5443 | * |
| hsa-miR-6087 | 1.10726 | ** |
| **Down-regulated miRNAs** | | |
| hsa-miR-3193 | -4.494046 | ** |
| hsa-miR-155-5p | -3.110721 | ** |
| hsa -miR-210 | -2.295794 | ** |
| hsa-miR-383 | -2.163116 | ** |
| hsa-miR-1249 | -2.057886 | ** |
| hsa-miR-1291 | -1.970409 | ** |
| hsa-miR-450a-3p | -1.877283 | * |
| hsa-miR-4750-5p | -1.877283 | * |
| hsa-miR-4435 | -1.410882 | ** |
| hsa-miR-652-5p | -1.362785 | * |
| hsa-miR-3676-3p | -1.333007 | * |
| hsa-miR-324-5p | -1.292281 | ** |
| hsa-let-7a-3p | -1.255735 | ** |
| hsa-miR-548l | -1.243389 | ** |
| hsa-miR-664a-3p | -1.238833 | ** |
| hsa-miR-4745-5p | -1.21426 | ** |
| hsa-miR-590-3p | -1.179775 | ** |
| hsa-miR-4466 | -1.107786 | * |
| hsa-miR-5189 | -1.095764 | * |
| hsa-miR-1277-5p | -1.082217 | ** |
| hsa-miR-1303 | -1.063451 | * |
| hsa-miR-122-5p | -1.052785 | * |
| hsa-miR-21-3p | -1.051488 | ** |
| hsa-miR-15b-3p | -1.016119 | ** |

**P*<0.05;***P*<0.01
