# Supplementary material for: MCRS1 overexpression, which is specifically inhibited by miR-129*, promotes the epithelial-mesenchymal transition and metastasis in non-small cell lung cancer
Source: Mol Cancer. 2014 Nov 6;13:245. doi: 10.1186/1476-4598-13-245 (PMC4233086; doi:10.1186/1476-4598-13-245)
Supplement: Supplementary file 6 — Additional file 6: Differentially expressed miRNAs in EPLC-32 M1 and MCRS1-depleted EPLC-32 M1 cells: novel miRNAs. (DOC 50 KB) [file 12943_2014_1444_MOESM6_ESM.doc]

**Additional file 6: Differentially expressed miRNAs in EPLC-32M1 and MCRS1-depleted EPLC-32M1 cells: novel miRNAs.**

| **Name** | **Fold change**  **Log2>=1** | **Sig-lable of P value** |
| --- | --- | --- |
| **Up-regulated miRNAs** | | |
| novel_mir_25 | 16.030798 | ** |
| novel_mir_150 | 7.7552882 | ** |
| novel_mir_137 | 7.7046645 | ** |
| novel_mir_149 | 7.6521997 | ** |
| novel_mir_151 | 7.5411741 | ** |
| novel_mir_165 | 7.5411741 | ** |
| novel_mir_26 | 7.2192653 | ** |
| novel_mir_133 | 6.9848172 | ** |
| novel_mir_15 | 6.8973615 | ** |
| novel_mir_155 | 6.8973615 | ** |
| novel_mir_138 | 6.8042601 | ** |
| novel_mir_160 | 6.7047337 | ** |
| **Down-regulated miRNAs** | | |
| novel_mir_49 | -15.9617676 | ** |
| novel_mir_61 | -8.84495678 | ** |
| novel_mir_100 | -8.45265309 | ** |
| novel_mir_128 | -8.11561593 | ** |
| novel_mir_8 | -7.725673 | ** |
| novel_mir_93 | -7.45261191 | ** |
| novel_mir_96 | -7.39128656 | ** |
| novel_mir_101 | -7.03760186 | ** |
| novel_mir_115 | -7.03760186 | ** |
| novel_mir_129 | -6.77452353 | ** |
| novel_mir_104 | -6.67511022 | ** |
| novel_mir_130 | -6.67511022 | ** |
| novel_mir_131 | -6.67511022 | ** |
| novel_mir_63 | -6.67511022 | ** |
| novel_mir_83 | -1.47292385 | * |
| novel_mir_78 | -1.42099553 | ** |
| novel_mir_17 | -1.41774134 | ** |

**P*<0.05;***P*<0.01
