# Supplementary material for: MCRS1 overexpression, which is specifically inhibited by miR-129*, promotes the epithelial-mesenchymal transition and metastasis in non-small cell lung cancer
Source: Mol Cancer. 2014 Nov 6;13:245. doi: 10.1186/1476-4598-13-245 (PMC4233086; doi:10.1186/1476-4598-13-245)
Supplement: Supplementary file 9 — Additional file 9: The seven differentially expressed miRNAs selected through the integrated analysis of miRNA expression profiles with miRNA target prediction. (DOC 37 KB) [file 12943_2014_1444_MOESM9_ESM.doc]

**Additional file 9: The seven differentially expressed miRNAs selected through the integrated analysis of miRNA expression profiles with miRNA target prediction.**

| **Name** | **Fold change Log 2 cancer cell line/normal cell line** | | | **Sig-lable** |
| --- | --- | --- | --- | --- |
|  | EPLC-32M1/16HBE | 801D/16HBE | A549/16HBE |  |
| hsa-miR-129-1-3p | -9.10962171 | -3.80219322 | -6.33985 | ** |
| hsa-miR-1254 | 2.45323198 | 1.03143905 | 1.50287747 | ** |
| hsa-miR-1275 | 1.26192079 | 3.14277907 | 2.74819018 | ** |
| hsa-miR-1287 | 1.96000566 | -2.78016691 | 1.00337705 | ** |
| hsa-miR-1299 | -1.78268897 | -6.97211822 | 3.08230974 | * |
| hsa-miR-1303 | 3.21752105 | 3.79486612 | 2.76267606 | ** |
| hsa-miR-378a-3p | 2.03473250 | 2.44476122 | 1.63756763 | ** |
