# Supplementary material for: MCRS1 overexpression, which is specifically inhibited by miR-129*, promotes the epithelial-mesenchymal transition and metastasis in non-small cell lung cancer
Source: Mol Cancer. 2014 Nov 6;13:245. doi: 10.1186/1476-4598-13-245 (PMC4233086; doi:10.1186/1476-4598-13-245)
Supplement: Supplementary file 11 — Additional file 11: The cell lines used in this study. (DOC 34 KB) [file 12943_2014_1444_MOESM11_ESM.doc]

**Additional file 11. The cell lines used in this study**

| **Name** | **Cell type** | **Culture media** | **Resource** |
| --- | --- | --- | --- |
| 801D | Large cell lung cancer | RPMI 10%FBS | Cell Bank of Chinese Academy of Science |
| SPC-A-1 | Lung adenocarcinoma | RPMI 10%FBS | Cell Bank of Chinese Academy of Science |
| GLC-82 | Lung adenocarcinoma | RPMI 10%FBS | Cell Bank of Chinese Academy of Science |
| EPLC-32M1 | Lung squamous carcinoma | RPMI 10%FBS | German Cancer Research Center |
| A549 | Lung adenocarcinoma | RPMI 10%FBS | American Type Culture Collection |
| NCI-H292 | Mucoepidermoid lung  carcinoma | RPMI 10%FBS | American Type Culture Collection |
| 16HBE | Immortalized human bronchial epithelial cell | DMEM 10%FBS | Cell Bank of the Peking Union Medical College |
| PT67 | Retrovirus packing cell | DMEM 10%FBS | Clontech |
