# Supplementary material for: MCRS1 overexpression, which is specifically inhibited by miR-129*, promotes the epithelial-mesenchymal transition and metastasis in non-small cell lung cancer
Source: Mol Cancer. 2014 Nov 6;13:245. doi: 10.1186/1476-4598-13-245 (PMC4233086; doi:10.1186/1476-4598-13-245)
Supplement: Supplementary file 12 — Additional file 12: The primers used in this study. (DOC 41 KB) [file 12943_2014_1444_MOESM12_ESM.doc]

**Additional file 12: The primers used in this study.**

| **Plasmid construction** | **Forward primers (5'-3');**  **Reverse primers (5'-3')** |
| --- | --- |
| MCRS1 3'UTR | CGACGCGT AGAGCAGCCTGGCAAAATCT;  GGAAGATCTCTTTGGATACCTTCTTCTTCT |
| MCRS1 shRNA3 | GATCCGCTGAAGAACAACGGTGATTTCAAGAGAATCACCGTTGTTCTTCAGCTTTTTTG;  AATTCAAAAAAGCTGAAGAACAACGGTGATTCTCTTGAAATCACCGTTGTTCTTCAGCG |
| **mRNA expression analysis** | **Forward primers (5'-3');**  **Reverse primers (5'-3')** |
| Snail1 | TCGGAAGCCTAACTACAGCGA;  AGATGAGCATTGGCAGCGAG |
| Slug | TGTGACAAGGAATATGTGAGCC;  TGAGCCCTCAGATTTGACCTG |
| E-cadherin | ATTTTTCCCTCGACACCCGAT;  TCCCAGGCGTAGACCAAGA |
| ZO-1 | AGTAAGTCGTCCTGATCCTGAA;  TCGGCCAAATCTTCTCACTCC |
| DSG2 | ACGTAGAAGTTACGCGCATAAA;  GGGTCACAATTCCTTCGTTAGTT |
| ABCB1 | GGGAGCTTAACACCCGACTTA  GCCAAAATCACAAGGGTTAGCTT |
| GAPDH | TGTTGCCATCAATGACCCCTT;  CTCCACGACGTACTCAGCG |
| **miRNAs expression analysis** | **Forward primers ((5'-3')** |
| miR-129* | GCGAAGCCCTTACCCCAAA |
| miR-210 | TGTGACAGCGGCTGAAA |
| **ChIP-PCR** | **Forward primers ((5'-3');**  **Reverse primers ((5'-3')** |
| miR-155 promoter | GATCTGGAAAGCAGGGAGAC；  CCAGATGAGGAAACTGAGCA |

1: For miRNAs expression analysis, forward primers of miR-383, miR-1299 miR-155 were purchased from Guangzhou RiboBio Co., Ltd；U6 and miRNA reverse primer were purchased from Qiagen, Hilden, Germany.

2: For ChIP-PCR, the primers specific for the GAPDH promoter were provided by ChIP kit.
