# Supplementary material for: MCRS1 overexpression, which is specifically inhibited by miR-129*, promotes the epithelial-mesenchymal transition and metastasis in non-small cell lung cancer
Source: Mol Cancer. 2014 Nov 6;13:245. doi: 10.1186/1476-4598-13-245 (PMC4233086; doi:10.1186/1476-4598-13-245)
Supplement: Supplementary file 13 — Additional file 13: The antibodies used in this study. (DOC 42 KB) [file 12943_2014_1444_MOESM13_ESM.doc]

**Additional file 13. The antibodies used in this study.**

| **Protein** | **Assay** | **Antibody** | **Origin** |
| --- | --- | --- | --- |
| MCRS1 | Western Blot | Rabbit polyclonal antibody | R36649; Sigma, St. Louis, MO, USA |
| MCRS1 | Chromatin immunoprecipitation | Mouse monoclonal antibody | SC-376569; Santa Cruz Biotechnology Inc, CA, USA |
| E-cadherin | Western Blot | Rabbit monoclonal antibody | #3195; Cell Signaling Technology, Danvers, MA, USA |
| ZO-1 | Western Blot | Mouse monoclonal antibody | 610966; BD Transduction Laboratories, San Jose, USA |
| Occludin | Western Blot | Rabbit polyclonal antibody | 13409-1-AP; Protein Tech Group, Chicago, USA |
| Vimentin | Western Blot | Mouse monoclonal antibody | SC-73259; Santa Cruz Biotechnology Inc, CA, USA |
| RhoA | Western Blot | Mouse monoclonal antibody | 240302; Cell Biolabs, San Diego, CA, USA |
| GAPDH | Western Blot | Mouse monoclonal antibody | G8795; Sigma, St. Louis, MO, USA |
| CD44 | Flow cytometric analysis | Mouse monoclonal antibody | 555478; BD Pharmingen, San Diego, CA, USA |
| F-actin | Immunofluorescence Staining | NONE | A12379; Invitrogen, Carlsbad, CA, USA |
